# Genomic comparison of esophageal squamous cell carcinoma and its precursor lesions by multi-region whole-exome sequencing

**DOI:** 10.1038/s41467-017-00650-0

**Published:** 2017-09-12

**Authors:** Xi-Xi Chen, Qian Zhong, Yang Liu, Shu-Mei Yan, Zhang-Hua Chen, Shan-Zhao Jin, Tian-Liang Xia, Ruo-Yan Li, Ai-Jun Zhou, Zhe Su, Yu-Hua Huang, Qi-Tao Huang, Li-Yun Huang, Xing Zhang, Yan-Na Zhao, Jin-Ping Yun, Qiu-Liang Wu, Dong-Xin Lin, Fan Bai, Mu-Sheng Zeng

**Affiliations:** 1 Biodynamic Optical Imaging Center (BIOPIC), School of Life Sciences, Peking University, Beijing, 100871 China; 2 State Key Laboratory of Oncology in South China, Collaborative Innovation Center for Cancer Medicine, Sun Yat-Sen University Cancer Center, Guangzhou, 510060 China; 3 Department of Pathology, Sun Yat-Sen University Cancer Center, Guangzhou, 510060 China; 4 State Key Laboratory of Molecular Oncology, Chinese Academy of Medical Science and Peking Union Medical College, Beijing, 100021 China

## Abstract

Esophageal squamous dysplasia is believed to be the precursor lesion of esophageal squamous cell carcinoma (ESCC﻿); however, the genetic evolution from dysplasia to ESCC remains poorly understood. Here, we applied multi-region whole-exome sequencing to samples from two cohorts, 45 ESCC patients with matched dysplasia and carcinoma samples, and 13 tumor-free patients with only dysplasia samples. Our analysis reveals that dysplasia is heavily mutated and harbors most of the driver events reported in ESCC. Moreover, dysplasia is polyclonal, and remarkable heterogeneity is often observed between tumors and their neighboring dysplasia samples. Notably, copy number alterations are prevalent in dysplasia and persist during the ESCC progression, which is distinct from the development of esophageal adenocarcinoma. The sharp contrast in the prevalence of the ‘two-hit’ event on *TP53* between the two cohorts suggests that the complete inactivation of *TP53* is essential in promoting the development of ESCC.

## Abstract (web summary)

The pathogenesis of oesophageal squamous cell carcinoma is a multi-step process but the genetic determinants behind this progression are unknown. Here the authors use multi-region exome sequencing to comprehensively investigate the genetic evolution of precursor dysplastic lesions and untransformed oesophagus.

## Introduction

Esophageal cancer is the 8th most common and 6th most lethal cancer, having a 5-year survival rate as low as 15–25%^1^. Esophageal squamous cell carcinoma (ESCC) is the dominant subtype of esophageal cancer worldwide, accounting for 90% of the cases all over the world^2^. A notably high incidence is observed in China^3^. The genomic landscape of ESCC has been characterized^4–8^, revealing that the most common alteration is the mutation of *TP53* through single-nucleotide variations and/or copy number losses. Other genes involved in cell cycle regulation, such as *RB1*, *CCND1*, *CDKN2A*, and *NFE2L2; NOTCH1*/*2* from the NOTCH pathway; and *MLL2* and *EP300* from the histone modifiers, are also frequently altered. In addition, large-scale chromosome amplifications at 3q, 5p, and 8q and deletions at 3p are prevalent in ESCC.

The pathogenesis of ESCC is believed to be a multi-step process. At the initial stage, squamous epithelial cells exhibit nuclear atypia and abnormal maturation but do not invade through the basement membrane. This stage is known as dysplasia^9^, and it is believed to be the precursor lesion of ESCC. From a genomic perspective, little is known about the evolving process from dysplasia to ESCC, especially how and at which stage the key carcinogenic events are acquired. Previous studies indicated that *TP53* is altered in some of the patients with dysplasia^10, 11^, and some copy number alteration (CNA) events, such as amplifications in 3q and 5p and losses in 3p, are also identified in a small dysplasia cohort^12^. However, the full spectrum of mutations and CNAs of dysplasia is poorly explored, and the genomic progression from dysplasia to tumor needs to be further characterized. Sampling both precursor lesions and tumors derived from the same patient is a practical way to portray the process of tumor evolution, because they share an identical germline background. Most recently, similar research strategies have been applied to the study of tumor evolution in esophageal adenocarcinoma (EAC), which is another subtype of esophageal cancer mainly identified in Caucasian populations. Ross-Innes et al.^13^ reported that Barrett’s esophagus (BE), the precursor lesion of EAC, is highly mutated and polyclonal, but the copy number states remain stable. Meanwhile, Stachler et al.^14^ noted that there might be two paths for BE to transform to EAC: one way is through a gradual loss of tumor suppressor genes followed by genomic instability and amplification of oncogenes; the other way starts with the inactivation of *TP53*, followed by whole-genome doubling, which facilitates genomic instability and oncogene activation.

ESCC, as the most prevalent subtype of esophageal cancer, is dissimilar to EAC in its pathogenesis, risk factors, and genomic landscape^8, 15, 16^. Yet, the evolution from squamous dysplasia to ESCC remains obscured, thus demanding comprehensive exploration of the underlying genetic ordering events. In this study, by applying whole-exome sequencing (WES) on multiple samples covering different stages from dysplasia to tumor derived from the same patient, we find dysplasia carries high mutation load and obvious heterogeneity exists between dysplasia and tumor. Notably, CNAs are prevalent in dysplasia and persist during the ESCC progression. Moreover, the ‘two-hit’ events on *TP53* are dominant in the dysplasia and carcinoma samples from the 45 ESCC patients, but rare in the dysplasia samples from the 13 tumor-free patients, suggesting that the full inactivation of *TP53* is essential in promoting the development of ESCC.

## Results

### Squamous dysplasia has similar mutational burden to that of ESCC

**Fig. 1:**
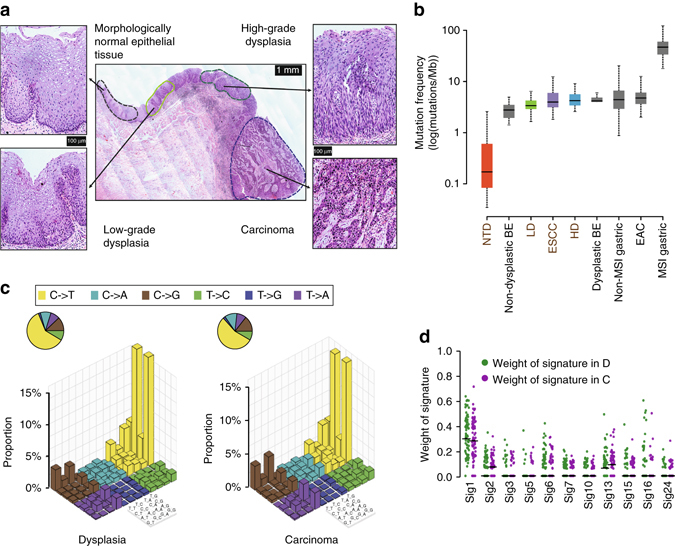
Mutational density and context of all samples. **a** Diagram showing the hematoxylin- and eosin-stained sections of morphologically normal epithelial tissue, low-grade dysplasia (LD), high-grade dysplasia (HD), and invasive esophageal squamous cell carcinoma (ESCC) from the same case. *Scale bars* are indicated. **b** Comparison of the mutational density of all NTDs (*n* = 13), LDs (*n* = 44), HDs (*n* = 31), and ESCCs (*n* = 62) with several other types of cancer. Non-dysplastic BE, non-dysplastic Barrett’s esophagus (*n* = 14); dysplastic BE, dysplastic Barrett’s esophagus (*n* = 11); EAC, esophageal adenocarcinoma (*n* = 183); MSI Gastric, microsatellite instable gastric cancer (*n* = 64); non-MSI Gastric, non-microsatellite instable gastric cancer (*n* = 231)^47^. The *y*-axis is shown on a log_10_-transformed scale. In each box plot, the lower and upper limits of the box denote the 25th (Q1) and 75th (Q3) percentiles, and the median value is indicated. The upper and lower whiskers show Q3 + 1.5 × IQR (interquartile range) and Q1−1.5 × IQR, respectively. **c** ‘Lego’ plots displaying the frequency of base substitutions within specific trinucleotide mutational contexts in all dysplasia samples (*left*) and ESCCs (*right*). **d** The weight of decomposed signatures in each of ESCCs (*purple dots*) and dysplasia samples (*green dots*). The mean value is indicated. C, ESCCs; D, dysplasia samples

We collected formalin-fixed paraffin-embedded (FFPE) tissue slides containing synchronously presented dysplasia regions and carcinoma regions from 45 ESCC patients (tumorous dysplasia cohort, TD cohort) as well as dysplasia samples from 13 patients with no sign of ESCC when endoscopic submucosal dissection or biopsy was performed (non-tumorous dysplasia cohort, NTD cohort; Supplementary Data 1). Laser capture microdissection (LCM) was applied to isolate different regions for WES (Fig. 1a). In the LCM process, all the samples were carefully dissected to avoid contamination of any immediate conjunct areas between two lesions. For 25 cases in the TD cohort, we obtained paired dysplasia and carcinoma samples. To further explore the potential heterogeneity and clonal progression, we managed to acquire additional 20 cases, in which multiple samples (3–9) covering tumor adjacent normal epithelial tissue, low-grade dysplasia (LD), high-grade dysplasia (HD), invasive carcinoma (ESCC), and metastasis were analyzed.

In total, 157 samples were sequenced, comprising 62 tumors (including 59 ESCCs and 3 lymph node metastases), 44 LD samples, 31 HD samples, 7 normal epithelial samples, and 13 dysplasia samples from the NTD cohort (Supplementary Fig. 1). An average sequencing depth of 83X was achieved for all samples (Supplementary Data 2).

For all ESCCs, the total mutation (silent and non-silent) number ranged from 53 to 507, with a median value of 80 (2.9 mutations/Mb) for non-silent mutations and 34 (1.1 mutations/Mb) for silent mutations, which is comparable to the results of previous studies (Fig. 1b)^4–8, 17^. In the TD cohort, the mutation density of HDs (4.1 mutations/Mb) was similar to that of ESCCs (3.9 mutations/Mb; Student’s *t*-test, *P* = 0.87), while the LDs had less mutations (3.3 mutations/Mb) than both HDs and ESCCs (Student’s *t*-test, *P* = 0.013 and *P* = 0.01, respectively). Both LDs and HDs had a higher mutation density than some malignancies, such as breast cancer (1.24 mutations/Mb)^18^. Interestingly, the dysplasia samples adjacent to ESCCs had a higher mutational density than those separate from ESCCs (Student’s *t*-test, *P* = 0.038; Supplementary Fig. 2a). Notably, the mutation density of LDs and HDs was comparable to that of BE, the precursor lesion in EAC^13, 14^. However, a significantly lower mutation density was observed in the NTDs (0.13 mutations/Mb). Since these lesions were also categorized as low-grade dysplasia (Supplementary Data 3) on histopathology, the smaller number of mutations accumulated compared to LDs in the TD cohort is possibly due to a shorter period after the initiation of neoplasia.

Regarding the mutational context, we observed a strong enrichment of C > T transitions in both squamous dysplasia and ESCC samples, especially at the CpG dinucleotides, which is denoted as signature 1 documented in the COSMIC Mutational Signature Framework (Fig. 1c, d), suggesting that the spontaneous deamination of cytosine contributes most to the accumulation of mutations during tumorigenesis^19^. Further comparison of dysplasia adjacent to ESCCs and separate from ESCCs in the TD cohort reported no significant difference in the mutational context (Supplementary Fig. 2b).

### Mutational landscape of squamous dysplasia and ESCC

**Fig. 2:**
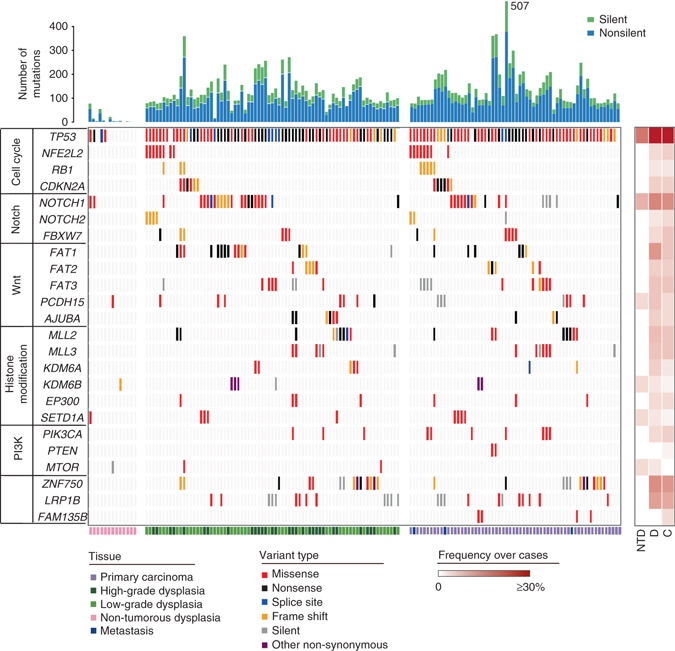
Mutational landscapes in the TD cohort and the NTD cohort. Somatic mutations in the TD and NTD cohort. *Top*, the number of silent and non-silent mutations of each sample. *Middle*, ESCC-associated genes in different pathways are ranked based on the mutation frequency. The heat map on the right indicates the frequency of each mutated gene in NTDs, dysplasia samples, and ESCCs over cases, respectively. *Bottom*, histopathological types of the samples in each case are indicated in different colors. Samples are displayed as columns

To compare the overall mutational landscape of squamous dysplasia and ESCC, we assessed the mutation frequency of ESCC-associated driver genes^4–8, 17^ in all samples from the TD and NTD cohorts. Generally, most of these driver genes were also present in dysplasia samples with high frequencies (Fig. 2). Mutations in *TP53* was the most recurrent event, present in dysplasia samples among 95.6% of patients and in ESCCs among 97.8% of patients. For EAC, although *TP53* is also found to be mutated in BE, its mutation frequency is low and is believed to be the boundary that distinguishes benign BE from dysplastic BE^20^. However, in ESCC we identified mutations in *TP53* with a high frequency in dysplasia samples (95.6%), and some were found even in NTDs (30.8%; Fig. 2), suggesting that *TP53* mutations may occur very early in ESCC progression. We also confirmed other frequently mutated driver genes, including *NOTCH1*, *ZNF750*, *FAT1*, *MLL2*, and *CDKN2A*, clustered in several ESCC-associated pathways, such as the NOTCH pathway, the Wnt pathway, cell cycle regulation, and histone modification, in dysplasia samples (Supplementary Fig. 3). It is noteworthy that genes within the NOTCH pathway exhibit an exclusive yet not statistically significant pattern, implying that these genes share overlapping functions. Taken together, these results show that dysplasia samples from the TD cohort share most of their mutational characteristics with matched ESCCs.

### Evident heterogeneity exists between dysplasia and ESCC samples

**Fig. 3:**
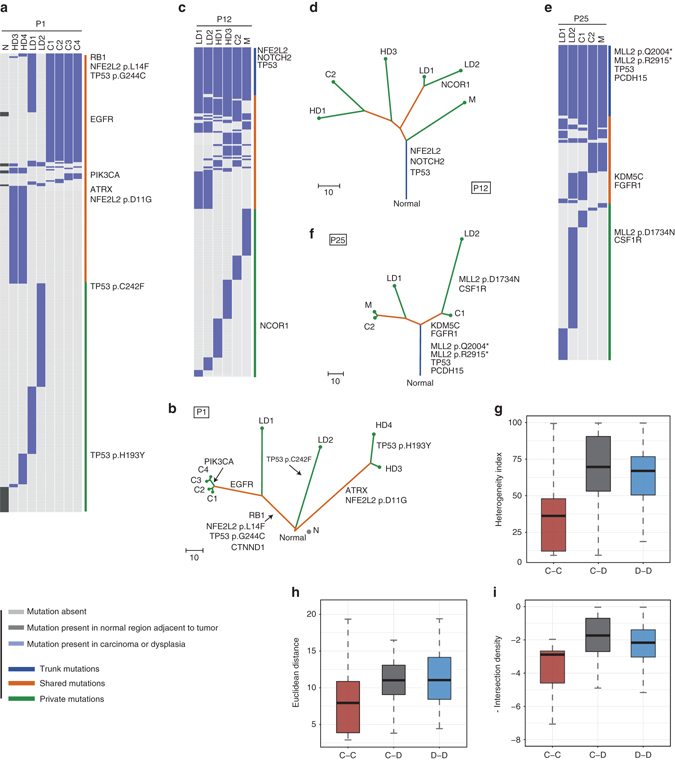
Regional distributions of somatic mutations and phylogenetic trees of typical cases. **a**–**f** Heat maps showing the regional distribution of all mutations among different samples (normal epithelial tissue, dysplasia and carcinoma) in each case. Mutations are further divided into three categories: present in all samples (*dark blue*), present in more than one but not all samples (*orange*), and present in only one sample (*green*). Phylogenetic trees are constructed based on the maximum parsimony algorithm for each case. The *color* of each line is corresponding to the categories of mutations shown in the heat map. The length of the trunk and branch is proportional to the number of mutations in each sample. There exist some mutations in the heat maps that do not fit the phylogenetic trees. The possible causes of this may be the absence of mutations due to copy number loss or geographically low level of clone intermixing, as well as other technical noise. **g**–**i** Box plots depicting the heterogeneity index (HI), the Euclidean distance and the density of intersecting mutations (in minus value) of pairwise samples in each case. All pairwise samples were divided into three groups: ESCC–ESCC (C-C), dysplasia-ESCC (C-D), and dysplasia–dysplasia (D-D). Kruskal–Wallis test, *P* = 4.50e^−07^ (HI), *P* = 0.00046 (Euclidean distance) and *P* = 2.45e^−06^ (-Intersection density)

For patients with multiple samples (≥3) from the TD cohort, we portrayed the regional distribution of mutations of dysplasia samples and ESCCs (Fig. 3a, c, e, Supplementary Fig. 4). Mutations were classified as ‘trunk’ if they were detected in all samples from one patient, ‘shared’ if they were detected in more than one but not all samples, and ‘private’ if they were specific to only one sample. Furthermore, we constructed phylogenetic trees to depict the clonal relationships and ordering events (Fig. 3b, d, f). In 4 of the 20 patients, the phylogenetic trees were barely rooted, with fewer than 7% mutations being “trunk”.

In P1, we obtained 4 ESCCs that were geographically distant from each other, as well as one normal epithelial tissue sample and 4 dysplasia samples (including 2 LDs and 2 HDs) surrounding the tumor (Supplementary Fig. 5a). The mutational burdens of the 4 dysplasia samples and 4 ESCCs were comparable, with an average of 100 mutations (Fig. 3a–f). The mutation distributions in the 4 ESCCs were highly similar, with 86.5% of the mutations present in all of them, indicating a monoclonal origin of the ESCCs in this patient. By contrast, of the 4 dysplasia samples, LD1 shared 42 mutations with the 4 ESCCs, including the well-known ESCC related genes *TP53*, *NFE2L2*, and *RB1*, while the LD2, HD3, and HD4 had nearly no overlapping mutation with all 4 ESCCs. To further resolve the clonal relationship of LD1 and matched ESCCs, we calculated the cancer cell fraction (CCF) of mutations in LD1 and the 4 ESCCs. By clustering the mutations according to their CCF values, we found several mutations that were clonal in ESCCs but subclonal in LD1 (Supplementary Fig. 5b). This finding strongly indicates that the 4 ESCCs were derived from a subclone of the LD1. Of the ESCC private mutations, we found *EGFR* (p.F712L) in all 4 ESCCs and *PIK3CA* (p.E545K) in C3 and C4, implying they occurred late during the tumorigenesis progression. Surprisingly, we observed remarkable heterogeneity within the dysplasia samples in P1. While LD2 harbored 97 mutations, only 4 of them were present in LD1. Although HD3 and HD4 shared 73 mutations, they did not overlap with LD1 or LD2. We also detected mutations in the histologically normal epithelial tissue in P1 (28 mutations; Fig. 3a). This finding supports the theory that mutations are already deposited in the histologically normal epithelial cells, as has recently been reported in other types of cancer, such as prostate carcinoma^21, 22^.

From the phylogenetic trees of P12 and P25 (Fig. 3d, f), each with one lymph node metastasis, we found that each patient’s samples had a common ancestor, with 29 trunk mutations in P12 and 47 trunk mutations in P25. Still, great heterogeneity was seen between dysplasia samples and ESCCs in these two patients. In P12, while possible driver mutations in *TP53*, *NOTCH2*, and *NFE2L2* were present on the trunk, all lesions were divergent from each other. Notably, we observed two distinct scenarios for the derivation of metastasis. In P25, metastasis had 91.4% of the mutations shared with one ESCC (C2), indicating that it was disseminated from C2 at a late stage. Through analysis of the CCF values of the mutations in P25_C2 vs. P25_M, we identified a cluster of mutations that are clonal in P25_M but subclonal in P25_C2, which also proved the relationship between the metastasis and C2 (Supplementary Fig. 6). By contrast, in P12, we found that the metastasis was the first to diverge on the phylogenetic tree. The CCF scatter plots of P12_M vs. any other sample showed a similar pattern. In any pair comparison, most shared mutations were clonal in both samples and these mutations were found to be located on the trunk after the manual inspection, suggesting that P12_M was related to other samples through a non-cancer ancestor. It was possible that the clone of the metastasis was derived early during the tumor evolution process, or because we failed to sample the related primary tumor clone of this metastasis.

Generally, all patients in the TD cohort showed branched clonal evolution. The proportion of trunk mutations ranged from 0 to 79.0%, with a median value of 21.0% (Fig. 3a–f, Supplementary Figs. 4 and 7), demonstrating an obvious heterogeneity in individual patients^23, 24^. Furthermore, we calculated the heterogeneity index (HI, the proportion of heterogeneous mutations relative to the total mutations of paired samples in each patient) in the dysplasia–dysplasia (D-D), dysplasia-ESCC (D-C), and ESCC–ESCC groups (C-C) from our TD cohort^25^ (Fig. 3g). The median HI of the C-C was 36.2%, which was comparable to the results of a recent study^17^. However, the highest HI was observed in the D-C (69.6%), while the HI in the D-D (67%) was also significantly higher than that in the C-C (Kruskal–Wallis test, *P* = 6.96e^−10^), and comparison of the Euclidean distance and the density of intersecting mutations of any paired samples in these three groups showed similar results (Fig. 3h, i). Consistently, we found that the dysplasia samples had significantly more subclonal mutations (Fisher’s exact test, *P* = 2.66e^−5^), strongly implying that the dysplasia samples were highly heterogeneous. In conclusion, the remarkable heterogeneity in the D-D and D-C groups revealed not all dysplasia belonged to the ESCC lineage, suggesting the divergent evolutionary paths taken by the precursor lesions and ESCCs^26^.

### Copy number alterations occur early in ESCC development

**Fig. 4:**
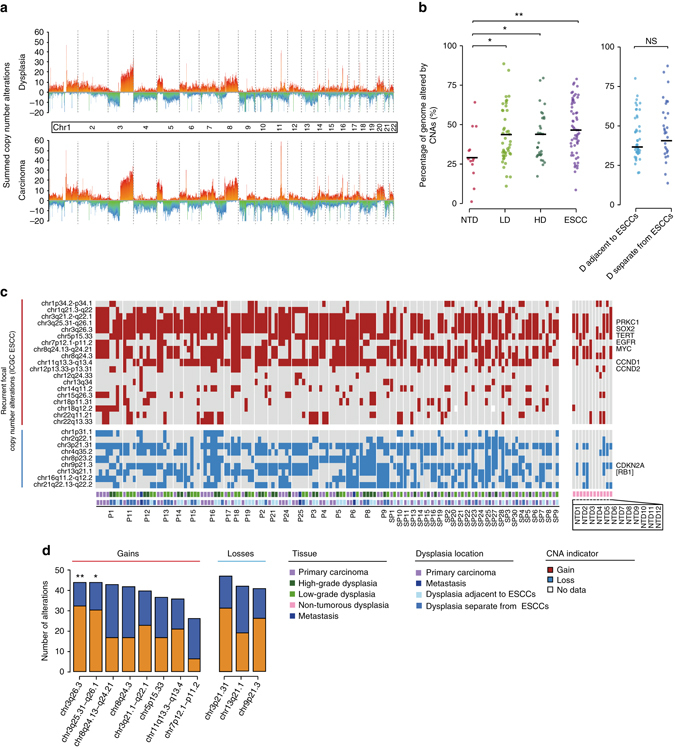
Copy number alterations in dysplasia samples and ESCCs. **a** Stacked mountain plots displaying the summed copy number alterations for 33 dysplasia samples (*top*) and 33 ESCCs (*bottom*). For each 500-Kb window of the whole genome, the depth ratios of all samples are transformed with log_2_ and summed. The copy number gains and losses are denoted by warm-color peaks and cool-color valleys, respectively. **b** *Left*, the fraction of genomic regions affected by CNAs in the four groups, NTDs, LDs, HDs, and ESCCs; *right*, the fraction of genomic regions affected by CNAs in the dysplasia adjacent to ESCCs and separate from ESCCs. The median value is indicated. Student’s *t*-test, *P < 0.05, **P < 0.01, NS, not significant. **c** Heat maps showing the recurrent CNAs reported in ICGC. Potential driver genes encompassed by certain cytobands are shown at right, and the square brackets represent the genes located adjacent to the cytoband. The results in the sample NTD13 is not shown due to the non-available data in these focal regions. **d** Bar plots showing the number of CNA events that are shared by all samples (*orange*) or part of samples (*blue*) in patients. Fisher’s exact test, *P < 0.05, **P < 0.01

Next, we evaluated the copy number states of the dysplasia samples and ESCCs using low-depth whole-genome sequencing (33 carcinoma samples and 33 dysplasia samples). Upon examination of the whole genome, both dysplasia samples and ESCCs showed extensive CNAs (Fig. 4a, Supplementary Fig. 8). Moreover, their copy number patterns displayed a high resemblance (Pearson correlation, *r* = 0.96; Supplementary Fig. 9), though such concordance was tilted to arm/chromosome level CNAs. To assess the CNAs more accurately, we also performed CNA calling based on the exome sequencing data, taking the purity and ploidy of each sample into account. The fractions of the genome affected by CNAs in the LDs, HDs, and ESCCs were similar. Meanwhile, no significant difference was found between dysplasia adjacent to ESCCs and dysplasia separate from ESCCs. However, the level of CNAs in the NTDs was significantly lower compared to that of samples in the TD cohort (Fig. 4b). We then examined the hot regions of CNAs previously reported in ESCC^4–8^, identifying gains in 3q (especially 3q25.31–3q26.1 and 3q26.3), 5p15.33, 7p12.1–7p11.2, 8q, and 11q13.3–11q13.4, as well as losses in 3p21.31, 9p21.3, and 13q21.1 with high frequencies in both dysplasia samples and ESCCs, and these recurrent CNAs tended to be shared by all samples in each patient (Fig. 4c). In addition, 8 of the dysplasia samples from the NTD cohort also harbored substantial CNAs (Fig. 4c, Supplementary Fig. 10). This evidence indicates that CNAs occurred at a relatively early stage of ESCC development, in contrast to EAC^13, 14^. In addition, gains in 3q and 5p as well as losses in 3p were also reported to be prevalent in head and neck, lung, and cervix squamous cell carcinoma^27, 28^, which highlights their essential functions in the development of squamous cell carcinoma. Specifically, gains in chromosome 3q (including 3q21.2–22.1, 3q25.31–26.1, and 3q26.3) existed in 43 of the 45 patients in the TD cohort, both in dysplasia samples and ESCCs. For those frequently altered regions in ESCC, we evaluated the proportion of the CNAs that were ubiquitous, and found that gains in 3q26.3 and 3q25.31–26.1 had significant bias towards being ‘trunk’ (Fisher’s exact test, *P* = 0.009 and *P* = 0.05, respectively; Fig. 4d). Shared alteration could result from early acquisition or convergent evolution. Of the 15 cases with the ubiquitous amplification in 3q, 11 cases possessed common breakpoints of the amplification events, which suggested the amplifications in 3q were obtained early. In addition, we observed that the gains in chromosome 3q existed in 8 NTDs. Interestingly, we also identified this alteration in the two histologically normal epithelial tissues in P1 and P5, while no other CNAs were identified across the genome (Supplementary Fig. 10, P1_N and P5_N). The 3q amplifications in NTDs and normal tissues were validated using qPCR (Supplementary Fig. 11). All these results indicated that amplifications in 3q may arise at the initial stage of malignant transformation.

**Fig. 5:**
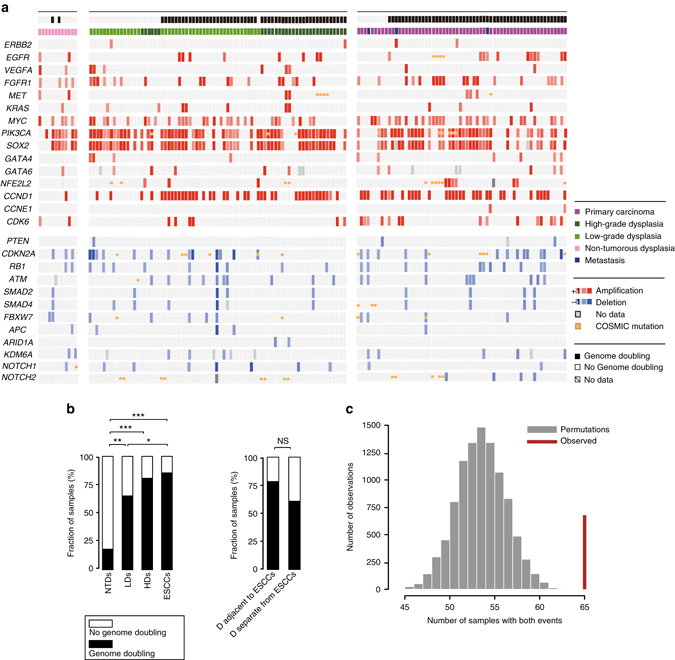
Comparison of the key alterations in dysplasia and ESCCs. **a** Plots showing the amplification of oncogenes (*red*), deletion of tumor suppressor genes (*blue*), and COSMIC mutations (*orange*) in NTDs (*left*), LDs/HDs (*middle*), and ESCCs (*right*). The genome doubling status and histopathological types of the samples were marked on the top. The results in the sample NTD13 is not shown due to the non-available data in these focal genes. **b** Comparison of the frequency of genome-doubling status in NTDs, dysplasia samples and ESCCs. Dysplasia samples adjacent to and separate from ESCCs in the TD cohort were also compared. Fisher’s exact test, *P < 0.05, **P < 0.01, ***P < 0.001, NS, not significant. **c** Permutation test of co-occurrence of genome doubling and *CCND1* amplification. The *gray* bars represent numbers of samples with co-occurred genome doubling event and *CCND1* amplification in permutations. The *red line* indicates the observed value in our data corresponding to an empirical *P* < 0.0001

Through the inspection of focal amplifications and deletions of driver genes, we observed a high occurrence of amplifications in *SOX2*, *PIK3CA*, *MYC*, *CCND1*, and *FGFR1* in both dysplasia samples and ESCCs, but the copy number status did not show obvious progression (Fig. 5a, Supplementary Fig. 12). We also checked the genome doubling events and found it prevalent in the TD cohort but not in the NTD cohort (Fig. 5b). Furthermore, within the TD cohort, the frequency of genome doubling events in LDs was significantly lower than that in ESCCs (Fisher’s exact test, *P* = 0.019), implying an increased genome instability along tumor progression. Notably, *CCND1* usually harbored high-level amplifications, which were enriched in samples with genome doubling events (Fig. 5c; Permutation test, *P* < 0.0001)^14, 29^.

We compared the heterogeneity of CNAs to that of mutations among all samples from the same patient, and found that the extent of heterogeneity of CNAs was generally consistent with that of mutations (Supplementary Fig. 13). Nevertheless, in several cases from the TD cohort (P6, P8, and P25), while the mutational distributions of the samples in each patient were dissimilar, we observed a high consistency in the copy number patterns, implying that in these patients, the CNAs not only emerged early but also remained steady during the ESCC progression.

### Two-hit event on *TP53* is essential for malignant transformation

**Fig. 6:**
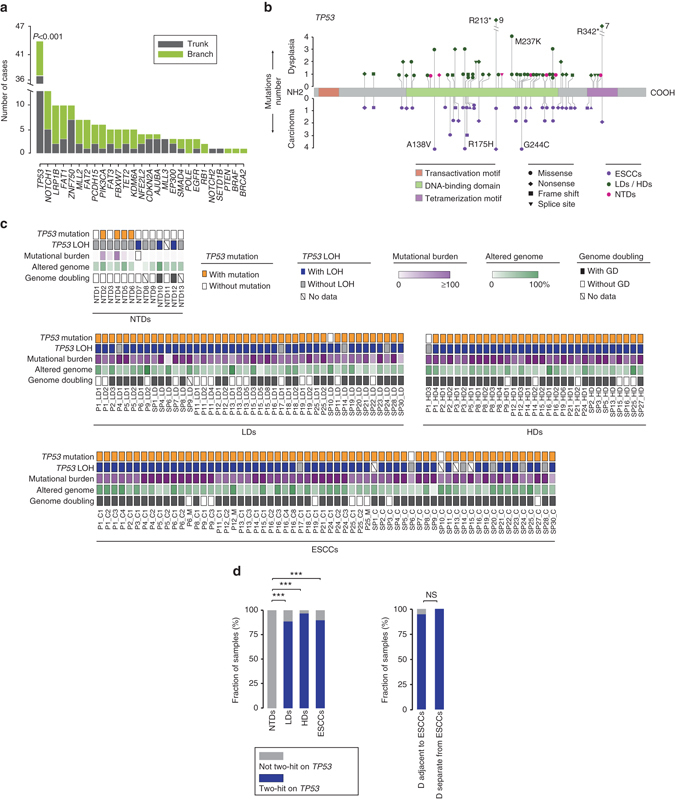
Mutation and loss of heterozygosity analysis of *TP53*. **a** Bar plots displaying the number of ‘trunk’ (*gray*) or ‘branch’ (*green*) mutations in indicated driver genes. The statistical significance is shown (Fisher’s exact test). **b** Schematics of protein changes in *TP53* identified in dysplasia samples (*top*) and ESCCs (*bottom*). Altered loci with high frequency are indicated. **c** Diagrams showing the *TP53* mutations, *TP53* loss of heterozygosity (LOHs), mutational burden, fraction of genome affected by CNAs, and genome doubling status. Each column represents a sample (sample name labeled below). **d** Comparison of the number of samples with or without the ‘two-hit’ event on *TP53* across three groups (NTDs, LDs, HDs, and ESCCs). Dysplasia samples adjacent to and separate from ESCCs in the TD cohort were also compared. Fisher’s exact test, ***P < 0.001, NS, not significant

For each ESCC-associated driver gene, we assessed the proportion of it as a truncal mutation across patients in our TD cohort, and identified that only *TP53* has significant bias towards being ‘trunk’ (Fig. 6a; Fisher’s exact test, *P* < 0.001). In 36 of the 42 cases, all samples shared at least one *TP53* mutation (Supplementary Fig. 14, Supplementary Data 6). In the other 6 cases, although each sample harbored one or more *TP53* mutations, none was ubiquitous among all samples. Moreover, the phylogenies of these 6 cases suggested that samples carrying independent *TP53* mutations came from distantly related clones. Nevertheless, according to the CCF, most of the mutations in *TP53* were clonal. In addition, 4 of the 13 NTDs possessed mutations in *TP53* (Fig. 2). Thus, we inferred that mutations in *TP53* arose early during ESCC development^30, 31^. Since most of the mutations occurred in the DNA binding domain of the p53 protein (Fig. 6b), we performed the immunohistochemistry (IHC) staining to explore the phenotypic effect of these mutations (Supplementary Figs. 15 and 16, Supplementary Data 6). In contrast to the truncating mutations, which would lead to the depletion of the protein, the missense mutations usually resulted in an enhanced expression of p53 protein (Supplementary Fig. 15)^32^. Interestingly, the ESCC in case SP11 (SP11_C) had a shared protein truncating *TP53* mutation and a private subclonal missense mutation. Our IHC results corroborated the mutational status at the protein level (Supplementary Fig. 16).

When we further considered the loss of heterozygosity (LOH) of *TP53*, we observed a remarkable difference between dysplasia samples from the TD cohort and those from the NTD cohort (Fig. 6c). In the TD cohort, 69 of the 71 dysplasia samples had a mutated *TP53* in conjunction with LOH (two-hit), which is similar to the results of ESCCs, in which 51 of the 54 samples harbored the two-hit alterations in *TP53*. Comparison of the LDs and HDs as well as dysplasia adjacent/non-adjacent to ESCCs reported no significant difference (Fig. 6d). In contrast, in the NTD cohort, even 3 NTDs showed LOH in *TP53*, none of the 10 NTDs harbored both the mutation and LOH in *TP53* (samples with more than 1 mutation in *TP53* but without LOH were excluded in the statistical analysis; Fig. 6d, Supplementary Fig. 17). In other words, most of the NTDs reserved at least one wild-type *TP53* allele, which could partially function to protect the cell from malignant transformation. A significantly lower mutational burden as well as less CNA-affected genome may also result from the fact that a wild-type *TP53* allele is reserved in NTDs. That may explain why only a small proportion of cases with dysplasia finally develop ESCC^9^. In conclusion, we inferred that LOH or mutations in *TP53* could either occur early but the further development of ESCC requires the full inactivation of *TP53*.

## Discussion

Our study, for the first time, portrayed the landscape of genomic alterations in the precursor lesions of ESCC and delineated clonal evolution in ESCC development. According to our data, the dysplasia neighboring to a tumor usually bears a high mutational burden, and most of the significantly mutated genes and recurrent CNAs in ESCC are also prevalent in tumor-neighboring dysplasia. However, a significantly lower number of mutations and fewer CNAs were observed in the dysplasia from our NTD cohort. One possible reason accounting for this scenario is that the NTDs undergo a shorter developmental period. The lower level of genomic variations detected could also result from the fact that the NTDs are highly polyclonal and lack of dominant clones where the genomic variations could be detected.

Despite comparable mutational burdens, the heterogeneity between dysplasia and ESCCs in each given patient is extensive. In fact, distinct evolution trajectories of precursor and tumors are also observed in other types of cancer, such as EAC and pancreatic carcinoma^13, 14, 26^. This gives us a hint that a diversified mutation background exists when the neoplasm initiates, conferring on the abnormal cells the ability to evade selection pressure, such as immune surveillance. Furthermore, we observed an early divergence of metastasis in one patient (P12), raising the possibility that this metastatic clone disseminated from the primary even before the primary became invasive. However, it is also probable that we failed to sample the invasive carcinoma region that gave rise to this metastasis.

In the present study, we found that the evident CNAs in the precursor lesions of ESCC are one of the major genetic features distinguishing it from EAC. The early emergence of CNAs was not only revealed by multi-region sequencing of both dysplasia samples and ESCCs from one patient in the TD cohort but also validated through the dysplasia samples from the NTD cohort. Particularly, in 3 patients (P6, P8, and P25), we observed discordant mutations but similar CNAs, suggesting the CNAs arose early and were maintained during the progression. In summary, we postulate that the accumulation of CNAs and mutations may represent two distinct genetic timers during tumorigenesis of ESCC.

Notably, even though dysplasia samples and ESCCs represent different histopathological stages, from the genetic perspective, we found these two groups in our TD cohort shared the same aberrant characteristics with each other. Most of the previously reported potential driver events for ESCC are also detected in dysplasia. However, the ordering events from dysplasia to carcinoma remain unclear. It may result from the “mini-driver” effect^33^, or intergenic and epigenetic alterations, which deserve further investigation. Alternatively, dysplasia neighboring to a tumor may have already gained transforming potential, but its growth is suppressed due to the competition to the dominant ESCC clone. Nonetheless, this result reminds us that the complete removal of tumors as well as dysplasia is highly necessary when resection or ablation is performed, and provides guidelines for the assignment of the resection margin.

By analyzing the truncal events across cases in the TD cohort, we speculated that mutations in *TP53* and gains in 3q are early alterations during the ESCC development. Moreover, we identified *TP53* mutations in 4 NTDs, while gains in 3q were present in 8 NTDs and 2 normal tissues adjacent to tumors, reinforcing the idea that they play essential roles in initiating the tumorigenesis. The higher frequency of gains in 3q observed in the early stage implies that it may occur prior to acquisition of *TP53* mutations. The sharp contrast in the frequency of two-hit events on *TP53* between the NTD cohort and the TD cohort suggests this event was necessary for transformation to malignancy. However, as LDs/HDs in the TD cohort also harbored high frequency of two-hit events on *TP53*, we could not claim that it was sufficient for the transformation. What blocked the phenotypic transition in LD/HD with two-hit on *TP53* remains cryptic in the current study.

ESCC and EAC are different cancer types originated from esophagus. As previously discussed by Stachler et al.^14^, one major path for BE to transform to EAC is through the inactivation of *TP53*, followed by whole-genome doubling. We looked for such evolutionary pattern in ESCC and also identified prevalence of *TP53* inactivation and genome doubling events in the TD cohort, which reminded us that there might be parallels in the general patterns of progression of these two cancer types. However, these two cancer types differed in the overall genomic landscapes^8^, and most importantly, in the timing of acquisition of CNAs. One limitation of our work is that we were not able to get the serial sampling of the lesions along disease progression of the same patient^13^, including normal squamous tissues and neoplasm samples from different stages, which is the ideal way to study tumor evolution. In conclusion, our work represents the beginning step of investigating the genetic relationship between ESCC and its precursor lesions. In the future, integrated multi-omics analysis will shed further light on the development of ESCC.

## Methods

### Sample collection and DNA extraction

This research was approved by the institutional review board of the Sun Yat-Sen University Cancer Center. Our study includes 45 patients with both ESCC and precursor lesions and 13 patients with only non-tumorous dysplasia lesions (Supplementary Data 1). All patients received no treatments before surgery and gave informed consent. FFPE samples were retrieved from the Tumor Resource Bank of Sun Yat-Sen University Cancer Center. The collection and publication of human genetic data has been approved by the Ministry of Science and Technology of China. Blood (48/58 patients) or normal esophageal tissues (10/58 patients) distant from lesion regions were used as germline controls. Slides stained with hematoxylin and eosin were reviewed independently by three experienced pathologists to identify the consensus areas of morphologically normal epithelial tissue (adjacent to tumor/dysplasia), LD, HD, and ESCC^34, 35^. The 3 pathologists first reviewed the cases independently, and their grading were collected. When their opinions were contradictory, they looked at the case together. If a consensus had been agreed, the samples were included in our study. Those samples that still remained controversial were excluded. In total, 49 cases in the TD cohort and 14 cases in the NTD cohort were reviewed and we excluded 5 of them from this study due to the disagreement of judgement. The moderate grading samples were included in the LD category in this work. Samples were cut into 5–10 consecutive 10-μm sections for LCM. A total of 157 distinct areas of interest were isolated via LCM using Leica LMD7000 Microsystem (Wetzlar; Supplementary Data 3). DNA was extracted using the QIAamp DNA Micro Kit (Qiagen) according to the manufacturer’s protocol with the exception that UNG enzyme was applied after decrosslinking to wipe out deaminated cytosines caused by formalin fixation.

### Whole-exome sequencing

For microdissected samples, an amount of 40–200 ng genomic DNA was sheared into fragments of 200–300 bp using the Covaris ultrasonic system (Covaris). The fragmented DNA was end-repaired, 5′-phosphorylated and ligated to barcoded sequencing adapters using a SPARK Lib Prep Kit (Enzymatics) according to the manufacturer’s protocol. For WES, the exonic regions of each sample were captured using a SureSelect V5 whole exon kit (Agilent). The product was quality checked and paired-end sequenced on Illumina Hiseq 2500 and 4000. A mean depth of 80X in the target region was achieved for all samples (Supplementary Data 2).

### Low-depth whole-genome sequencing

For whole-genome sequencing, libraries were constructed as described in the WES library preparation process with the exception of exome capture. The product was quality checked and paired-end sequenced on Illumina Hiseq 2500 and 4000. The mean sequencing depth is 0.5X for all samples.

### SNV and INDEL calling

Sequencing reads were aligned to the reference genome hg19 build (UCSC) using BWA (BWA-0.5.9) with default parameters to generate a binary sequence alignment map (BAM) file^36^. The aligned BAM file was sorted and merged using Samtools 0.1.19^37^, and the duplications were marked and removed using Picard tools. Then, realignment of all insertions and deletions (INDELs) and recalibration of base quality were done with Genome Analysis Toolkit (GATK2.1-8)^38^. For mutation calling, we first used the GATK Unified Genotyper in multi-sample mode to call single nucleotide variations (SNVs) and INDELs. Then we applied another joint-calling method MultiSNV^39^ for SNV calling and variants qualified in both methods were reserved. INDELs called by the GATK Unified Genotyper were manually checked to ensure the fidelity. All variants were annotated by SNPEFF 3.0^40^. We set the following criteria for identification of reliable somatic SNVs or INDELs: (1) reads covering the mutated sites should number more than 10, with at least 3 reads harboring the mutations; (2) reads covering the mutated sites in the corresponding normal control should number more than 10, with at most 1 read harboring the mutations; (3) mutations listed in dbSNP 135 were removed unless they were documented by the Catalog of Somatic Mutations in Cancer (COSMIC); and (4) mutations listed in the National Heart, Lung, and Blood Institute Exome Sequencing Project were excluded (Supplementary Data 4). After the standard calling, for variants detected in at least one but not all samples in each case, we adopted the “force-calling” strategy^14^ to improve the sensitivity of mutations calling by rescuing those variants missed in other samples due to low variant allele frequency (VAF).

To further confirm the fidelity of mutations that were called as described above, we validated a total of 96 non-silent mutations from 19 samples of 5 patients (SP6, SP14, P5, P11, and P14). Multiplex PCRs were performed on 10 ng genomic DNA from each sample (samples without additional DNA were not included) of one patient using the Multiplex PCR Kit according to the manufacturer’s protocol (Vazyme). The barcoded libraries were then constructed and sequenced on Illumina 4000. All mutations had a read coverage greater than 200, with a mean depth of 248X in target regions. Mutations with a VAF <0.02 were considered absent, while those with a VAF >0.02 in germline controls were considered as germline variants. A validation rate of 98.6% was achieved (Supplementary Data 5).

### Determination of potential driver mutations

To identify potential driver mutations in dysplasia samples and ESCCs, we evaluated the non-synonymous mutations from the following three aspects: (1) Mutations in significantly mutated genes highlighted by recent large cohort sequencing studies of ESCC^4–8, 17^; (2) Mutations in genes that are documented by the COSMIC database (ESCC-associated or related to other types of cancer); (3) Mutations in genes that are present in the KEGG (Kyoto Encyclopedia of Genes and Genomes) pathways in cancer. Putative driver mutations were determined if they matched one of the above requirements.

### Copy number analysis and LOH identification

We first used whole genome sequencing data of 67 samples (1 morphologically normal epithelial tissue, 33 dysplasia samples and 33 ESCCs) to portrait the copy number states upon the whole-genome view as described in our previous study^41^. In detail, we aligned sequencing reads to reference genome hg19 build (UCSC) using BWA (BWA-0.5.9) with default parameters to generate BAM files. After removing duplications, we split the genome into 500 Kb windows and summed the coverage depth of each window. The size of a diploid human genome should be 3 Gb. Thus, at a theoretical depth of 1X, the coverage of each window should equal to its size 500-Kb. We could calculate the copy number (CN) of each sample as follows:

$$\frac{{{\rm{CN}}}}{2} = \frac{{{\rm{Cov}}\left( {{\rm{Kb}}} \right){\rm{/Dat}}\left( {{\rm{Gb}}} \right)}}{{500\left( {{\rm{Kb}}} \right){\rm{/}}3\left( {{\rm{Gb}}} \right)}}$$

Where Cov is the coverage depth (Kb) observed of each window normalized by the CG content and Dat represents the total sequenced data volume (Gb). Next, we integrated these windows into segments based on the circular binary segmentation algorithm from DNAcopy package in R^42^.

To more accurately determine the allele-specific CN, we applied Sequenza R package 2.1.1 to evaluate CN states among all whole-exome sequenced samples^43^. Standard BAM files of samples with their matched normal controls were used as input to calculate the depth ratio considering both GC contents and data quantity. We further estimated the purity and ploidy of samples with following parameters: breaks.method = ‘full’, gamma = 40, kmin = 5, gamma.pcf = 200, kmin.pcf = 200. The processed segmented CN data of samples was divided by sample ploidy and transformed with log_2_ to identify CNAs. The cutoff values of gain and loss were defined as log_2_(2.5/2) and log_2_(1.5/2)^25^. All CNAs were compared with significantly altered regions reported in the International Cancer Genome Consortium (ICGC) data to assess the ubiquitous events in both dysplasia samples and ESCCs of patients. For each sample, the fraction of genome affected by CNAs was calculated by the length of amplified or deleted regions relative to the total length of the segments. Moreover, we summarized the CN of the major and minor allele (B allele) calculated by Sequenza. To identify the loss of heterozygosity (LOH) events, we inspected the BAF values of germline heterozygous SNPs. If there existed a LOH, we should observe the deviation of the BAF values from 0.5 (ignoring the allelic bias caused by the exome capture process). Additionally, ABSOLUTE^44^ was used to analyze our data to further confirm the validity of LOH calling. Meanwhile, genome doubling events were also identified.

### Phylogenetic tree construction

All qualified mutations (including silent and non-silent) of samples in 20 patients were used to construct the phylogenetic trees. In detail, sequences encompassing the mutations in the total length of 21 bp were extracted to infer the phylogeny among samples of each patient based on the maximum parsimony algorithm using MEGA 7.0.14 with default parameters^45^. Putative driver mutated genes were labeled on the trees according to their regional distributions. For the same gene with more than one mutation in a given patient, the specific amino acid changes were indicated. The additional morphologically normal epithelial tissues adjacent to tumors from 7 patients (P1, P3, P4, P5, P9, P15, and P17) were not included in the phylogenetic analysis.

### Evaluation of heterogeneity of mutations and CNAs

We first evaluated the heterogeneity of mutations for 45 patients with both dysplasia samples and ESCCs. Through pairwise comparisons of different samples in each case, mutations were classified into shared and private categories. For each pair of samples, we calculated the proportion of private mutations relative to the total number of mutations as HI^25^. All pairwise comparisons were performed across three groups: ESCC–ESCC (C-C), dysplasia-ESCC (C-D) and D–D. Through pairwise comparisons of different samples in each case, mutations were classified into shared and private categories. For each pair of samples (A and B), we calculated the proportion of private mutations relative to the total number of mutations as heterogeneity index (HI) by the following formula:

$${\rm{HI}} = 1 - \frac{{A \cap B}}{{A \cup B}}$$

where *A* and *B* denote the mutation sets of A and B, respectively. We also calculated the Euclidean distance and the density of intersecting mutations as other ways to evaluate the heterogeneity among dysplasia samples and tumor samples.

We next evaluated the heterogeneity of CNAs for 20 patients. Segments from CN profile with partial overlap among different samples within the same patient were split into short consecutive segments to obtain a file which contained only overlapping or non-overlapping segments between any two samples within one patient. The depth ratio of each segment was used as the input to calculate the Pearson correlation coefficient for evaluating the heterogeneity of CNAs.

### Mutational signature analysis

Base substitutions could be divided into 6 categories, namely, C > T, C > A, C > G, T > C, T > G, and T > A. Considering the 5′ and 3′ flanking nucleotides of a specific mutated base, a total of 96 substitution types exist. We first compared the proportion of mutations within specific contexts in dysplasia samples and ESCCs of 45 patients. To extract the underlying mutational signatures in single dysplasia samples and ESCCs, we then applied the R package deconstructSigs^46^ to each sample using the 30 signatures documented by the COSMIC as reference. After extraction, we calculated and compared the mean weights of different signatures in dysplasia samples and ESCCs. 3 samples with SNV number less than 50 were not included in this analysis.

### Calculation of cancer cell fraction

Inspired by the methods used in previous studies^23, 25, 44^, for each somatic mutation, we estimated the CCF by integrating the purity of the sample, the local integer CN and the CN of mutant allele. Given a certain CCF value of one mutation, we could calculate the expected VAF as follows:

$${\rm{VAFe}} = \frac{{p}\,{{*}\,\rm{CN{t}*CCF}}}{{{\rm{CN}\,{*}}\,p + 2 \,{*}\,\left( {1 - p} \right)}}$$

Where VAFe is the expected VAF, *p* is the estimated purity of the sample, and CN represents the local integer CN of this mutation. CNt refers to the CN of the allele carrying the mutation, thus is either the major or the minor CN of the location. The mixture of normal diploid cells was taken into account, and 2 was set as the local CN of the matched normal sample (sex chromosomes were excluded in this analysis). Next, we compared the expected VAF (VAFe) to the observed VAF (VAFo) using a binomial test as follows:

$$P\left( {{\rm{CCF}}} \right) = {\rm{dbinom}}\left( {A|N,\,{\rm{VAFe}}\left( {{\rm{CCF}}} \right)} \right)$$

In this formula, *A* represents the number of altered reads and *N* is the depth at this mutated site. Then, we could calculate the probability distribution of 100 CCF values from 0.01 to 1 and get the most likely CCF. Mutation with a CCF value <0.8 was considered as a subclonal mutation.

### Validation of chr3q amplifications by qPCR

For 4 NTDs and 2 two histologically normal epithelial tissues (P1_N and P5_N) with additional genomic DNA (gDNA), the quantitative PCR was performed using the Sybr Green mix kit (Vazyme) on the Bio-Rad CFX96 system (Bio-Rad). Primers designed for target region within *SOX2* gene on cytoband 3q26.3 were: forward: 5′-CTCTTGGCTCCATGGGTTC-3′ and reverse: 5′-GGAGTGGGAGGAAGAGGTAA-3′. *RPPH1* gene was used as neutral CN control (primers: forward: 5′-GGAGCTTGGAACAGACTCAC-3′ and reverse: 5′-GGAGAGTAGTCTGAATTGGGTTATG-3′). 5 blood gDNA samples were used as negative control and gDNA extracted from 3 ESCCs samples with amplifications in 3q26.3 were used as positive control. The experiments of each sample were repeated 3 times, and the results were analyzed using ΔΔCt method.

### Immunohistochemistry staining analysis

The 4-μm FFPE samples were placed in an oven for 2 h at 65 °C, and then deparaffinized in xylenes and rehydrated using an ethanol alcohol gradient followed by distilled water. The sections were immersed in 3% hydrogen peroxide for 10 min to block endogenous peroxidase activity at room temperature. After that, the sections were treated with Tris-EDTA Antigen Retrieval Solution (pH = 9.0) for 5 min in a pressure cooker for antigen retrieval. After the temperature of the antigen retrieval solution returned to room temperature, the sections were incubated with the mouse monoclonal anti-p53 antibody (diluted 1:50, Gene Tech Company Limited, Shanghai, China) at 37 °C for 1 h in a moist chamber and a biotinylated goat anti-mouse antibody (DAKO, Santa Clara, CA, USA) at 37 °C for 30 min on the next day. Subsequently, the sections were stained for protein detection in DAKO Liquid 3,′3-diaminobenzidine tetrahydrochloride (DAB) (DAKO) and counterstained with Mayer’s hematoxylin, then were dehydrated and mounted.

### Statistical analysis

All statistical analysis was conducted using R v3.3.1 (foundation for statistical computing). The Student’s *t*-test was used to compare the significant differences between two groups in mutation density and fraction of genome affected by CNAs. The equality of variances was checked before the Student’s *t*-test was performed. The Fisher’s exact test was used to determine whether a potential driver alteration has bias towards being ‘trunk’, to compare the enrichment in the number of cases harboring mutations in ESCC-associated genes of different pathways, to compare the significant differences in the number of cases harboring the genome doubling event and the ‘two-hit’ event on *TP53*. The nonparametric Kruskal–Wallis test was used to compare the HI and Euclidean distance of pairwise samples across the three groups. No statistical methods were used to determine sample size. No samples were excluded from the present study. The experiments were not randomized and the investigators were not blinded to allocation during experiments and outcome assessment.

### Data availability

The WES and low-depth whole-genome sequencing data has been deposited in the database of NCBI Sequence Read Archive under accession number SRP099292. The detailed information of matched samples and patients are listed in the Supplementary Data 1 and 3. Publically available databases or resources used in this research are as follow: Picard (http://broadinstitute.github.io/picard/), dbSNP (http://www.ncbi.nlm.nih.gov/projects/SNP/), ESP (https://esp.gs.washington.edu/drupal/), COSMIC (http://cancer.sanger.ac.uk/cancergenome/projects/cosmic/), KEGG (http://www.kegg.jp/). All other remaining data are available within the Article and Supplementary Files, or available from the authors upon request.

## Electronic supplementary material

Supplementary Information

Supplementary Data 1

Supplementary Data 2

Supplementary Data 3

Supplementary Data 4

Supplementary Data 5

Supplementary Data 6

## References

[1] Pennathur A, Gibson MK, Jobe BA, Luketich JD (2013). Oesophageal carcinoma. The Lancet.

[2] Rustgi AK, El-Serag HB (2014). Esophageal carcinoma. N. Engl. J. Med..

[3] Stewart, B. W. & Wild, C. P. (eds) *World Cancer Report 2014* (IARC Press, 2014).

[4] Song Y (2014). Identification of genomic alterations in oesophageal squamous cell cancer. Nature.

[5] Lin DC (2014). Genomic and molecular characterization of esophageal squamous cell carcinoma. Nat. Genet..

[6] Gao YB (2014). Genetic landscape of esophageal squamous cell carcinoma. Nat. Genet..

[7] Zhang L (2015). Genomic analyses reveal mutational signatures and frequently altered genes in esophageal squamous cell carcinoma. Am. J. Hum. Genet..

[8] Cancer Genome Atlas Research, N (2017). Integrated genomic characterization of oesophageal carcinoma. Nature.

[9] Taylor PR, Abnet CC, Dawsey SM (2013). Squamous dysplasia--the precursor lesion for esophageal squamous cell carcinoma. Cancer Epidemiol. Biomarkers Prev..

[10] Kawakubo H (2005). Alterations of p53, cyclin D1 and pRB expression in the carcinogenesis of esophageal squamous cell carcinoma. Oncol. Rep..

[11] Gao H (1994). p53 tumor suppressor gene mutation in early esophageal precancerous lesions and carcinoma among high-risk populations in henan, China. Cancer Res..

[12] Shi ZZ (2013). Consistent and differential genetic aberrations between esophageal dysplasia and squamous cell carcinoma detected by array comparative genomic hybridization. Clin. Cancer Res..

[13] Ross-Innes CS (2015). Whole-genome sequencing provides new insights into the clonal architecture of Barrett’s esophagus and esophageal adenocarcinoma. Nat. Genet..

[14] Stachler MD (2015). Paired exome analysis of Barrett’s esophagus and adenocarcinoma. Nat. Genet..

[15] Dulak AM (2013). Exome and whole-genome sequencing of esophageal adenocarcinoma identifies recurrent driver events and mutational complexity. Nat. Genet..

[16] Bandla S (2012). Comparative genomics of esophageal adenocarcinoma and squamous cell carcinoma. Ann. Thorac. Surg..

[17] Hao JJ (2016). Spatial intratumoral heterogeneity and temporal clonal evolution in esophageal squamous cell carcinoma. Nat. Genet..

[18] Banerji S (2012). Sequence analysis of mutations and translocations across breast cancer subtypes. Nature.

[19] Alexandrov LB (2013). Signatures of mutational processes in human cancer. Nature.

[20] Weaver JM (2014). Ordering of mutations in preinvasive disease stages of esophageal carcinogenesis. Nat. Genet..

[21] Martincorena I (2015). Tumor evolution. High burden and pervasive positive selection of somatic mutations in normal human skin. Science.

[22] Cooper CS (2015). Analysis of the genetic phylogeny of multifocal prostate cancer identifies multiple independent clonal expansions in neoplastic and morphologically normal prostate tissue. Nat. Genet..

[23] Faltas BM (2016). Clonal evolution of chemotherapy-resistant urothelial carcinoma. Nat. Genet..

[24] de Bruin EC (2014). Spatial and temporal diversity in genomic instability processes defines lung cancer evolution. Science.

[25] Murugaesu N (2015). Tracking the genomic evolution of esophageal adenocarcinoma through neoadjuvant chemotherapy. Cancer Discov..

[26] Notta F (2016). A renewed model of pancreatic cancer evolution based on genomic rearrangement patterns. Nature.

[27] The Cancer Genome Atlas, N (2015). Comprehensive genomic characterization of head and neck squamous cell carcinomas. Nature.

[28] Cancer Genome Atlas Research, N (2012). Comprehensive genomic characterization of squamous cell lung cancers. Nature.

[29] Wang Q (2015). An old story retold: loss of G1 control defines a distinct genomic subtype of esophageal squamous cell carcinoma. Genomics Proteomics Bioinformatics.

[30] Durinck S (2011). Temporal dissection of tumorigenesis in primary cancers. Cancer Discov..

[31] Gibson WJ (2016). The genomic landscape and evolution of endometrial carcinoma progression and abdominopelvic metastasis. Nat. Genet..

[32] Kim JY (2016). Association between mutation and expression of TP53 as a potential prognostic marker of triple-negative breast cancer. Cancer Res. Treat..

[33] Castro-Giner F, Ratcliffe P, Tomlinson I (2015). The mini-driver model of polygenic cancer evolution. Nat. Rev. Cancer.

[34] DeNardi, F. G. & Riddell, R. H. *Esophagus in Histology for Pathologists* 3rd edn. (ed. Mills, S. E.) (Lippincott Williams & Wilkins: Philadelphia, 2007).

[35] Weinstein WM, Bogoch ER, Bowes KL (1975). The normal human esophageal mucosa: a histological reappraisal. Gastroenterology.

[36] Li H, Durbin R (2009). Fast and accurate short read alignment with Burrows-Wheeler transform. Bioinformatics.

[37] Li H (2009). The Sequence Alignment/Map format and SAMtools. Bioinformatics.

[38] McKenna A (2010). The Genome Analysis Toolkit: a MapReduce framework for analyzing next-generation DNA sequencing data. Genome Res..

[39] Josephidou M, Lynch AG, Tavare S (2015). multiSNV: a probabilistic approach for improving detection of somatic point mutations from multiple related tumour samples. Nucleic Acids Res..

[40] Cingolani P (2012). A program for annotating and predicting the effects of single nucleotide polymorphisms, SnpEff: SNPs in the genome of Drosophila melanogaster strain w1118; iso-2; iso-3. Fly (Austin).

[41] Ni X (2013). Reproducible copy number variation patterns among single circulating tumor cells of lung cancer patients. Proc. Natl Acad. Sci. USA.

[42] Venkatraman ES, Olshen AB (2007). A faster circular binary segmentation algorithm for the analysis of array CGH data. Bioinformatics.

[43] Favero F (2015). Sequenza: allele-specific copy number and mutation profiles from tumor sequencing data. Ann. Oncol..

[44] Carter SL (2012). Absolute quantification of somatic DNA alterations in human cancer. Nat. Biotechnol..

[45] Tamura K (2011). MEGA5: molecular evolutionary genetics analysis using maximum likelihood, evolutionary distance, and maximum parsimony methods. Mol. Biol. Evol..

[46] Rosenthal R, McGranahan N, Herrero J, Taylor BS, Swanton C (2016). DeconstructSigs: delineating mutational processes in single tumors distinguishes DNA repair deficiencies and patterns of carcinoma evolution. Genome Biol..

[47] Cancer Genome Atlas Research, N (2014). Comprehensive molecular characterization of gastric adenocarcinoma. Nature.

