# Supplementary material for: Genomic comparison of esophageal squamous cell carcinoma and its precursor lesions by multi-region whole-exome sequencing
Source: Nat Commun. 2017 Sep 12;8:524. doi: 10.1038/s41467-017-00650-0 (PMC5595870; doi:10.1038/s41467-017-00650-0)
Supplement: Supplementary file 1 — Supplementary Information [file 41467_2017_650_MOESM1_ESM.pdf]

Description of Supplementary Files

File Name: Supplementary Information
Description: Supplementary Figures

File Name: Supplementary Data 1
Description: Clinical information of all 58 patients

File Name: Supplementary Data 2
Description: Sequencing information of all samples of 58 patients

File Name: Supplementary Data 3
Description: Stage of each sample and number of somatic mutations

File Name: Supplementary Data 4
Description: Summary of somatic mutations of all 158 samples in 58 patients

File Name: Supplementary Data 5
Description: Validation of mutations by target sequencing

File Name: Supplementary Data 6
Description: Summary of all non-synonymous mutations in TP53

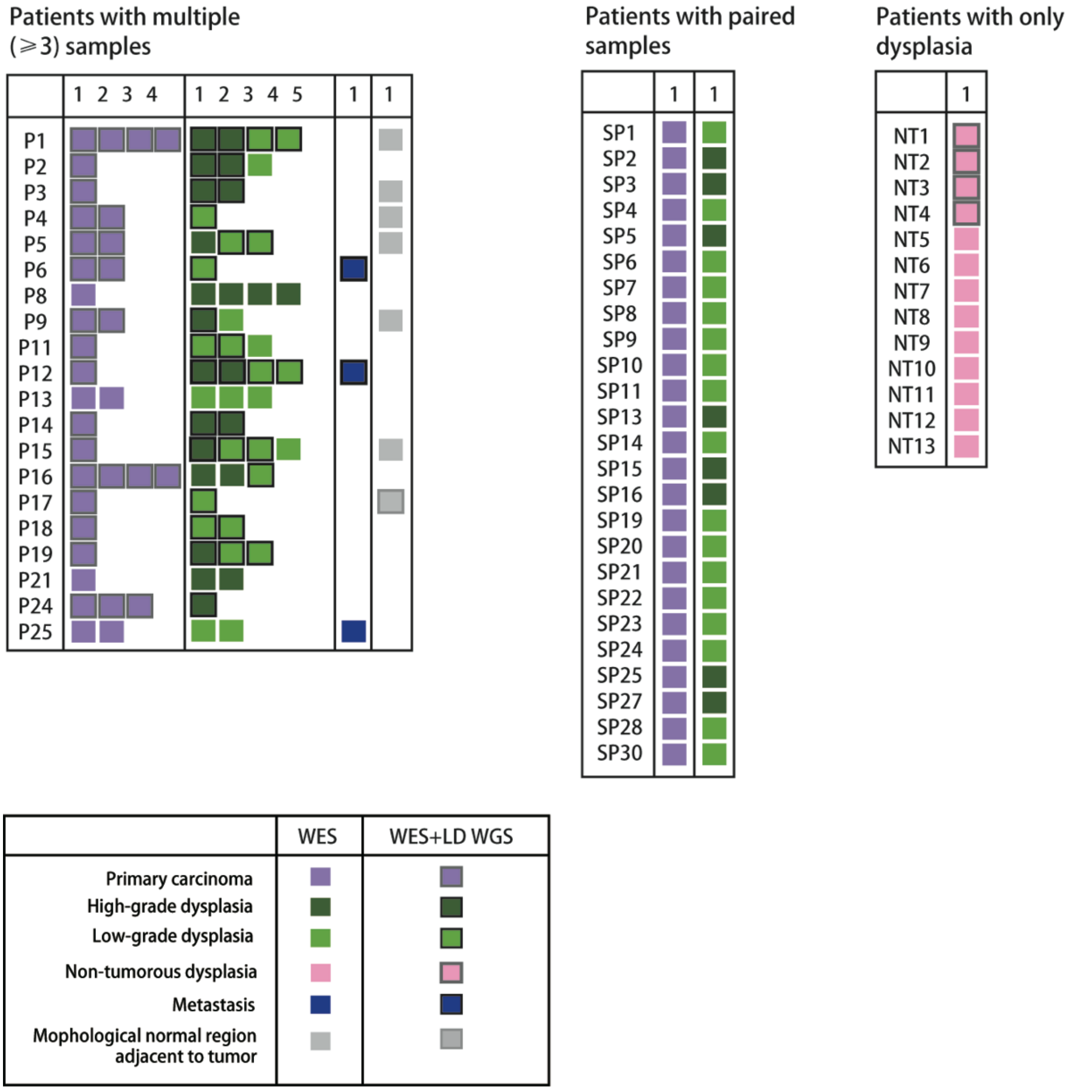

Supplementary Figure 1. Summary of sample set in this study. Patients with more than two samples (left), with paired samples (middle) from the TD cohort and patients with only dysplasia samples from the NTD cohort (right). All the samples underwent the whole exome sequencing (WES). Besides whole exome sequencing, 67 samples (with black border) were subjected to the low-depth whole genome sequencing (LD WGS).

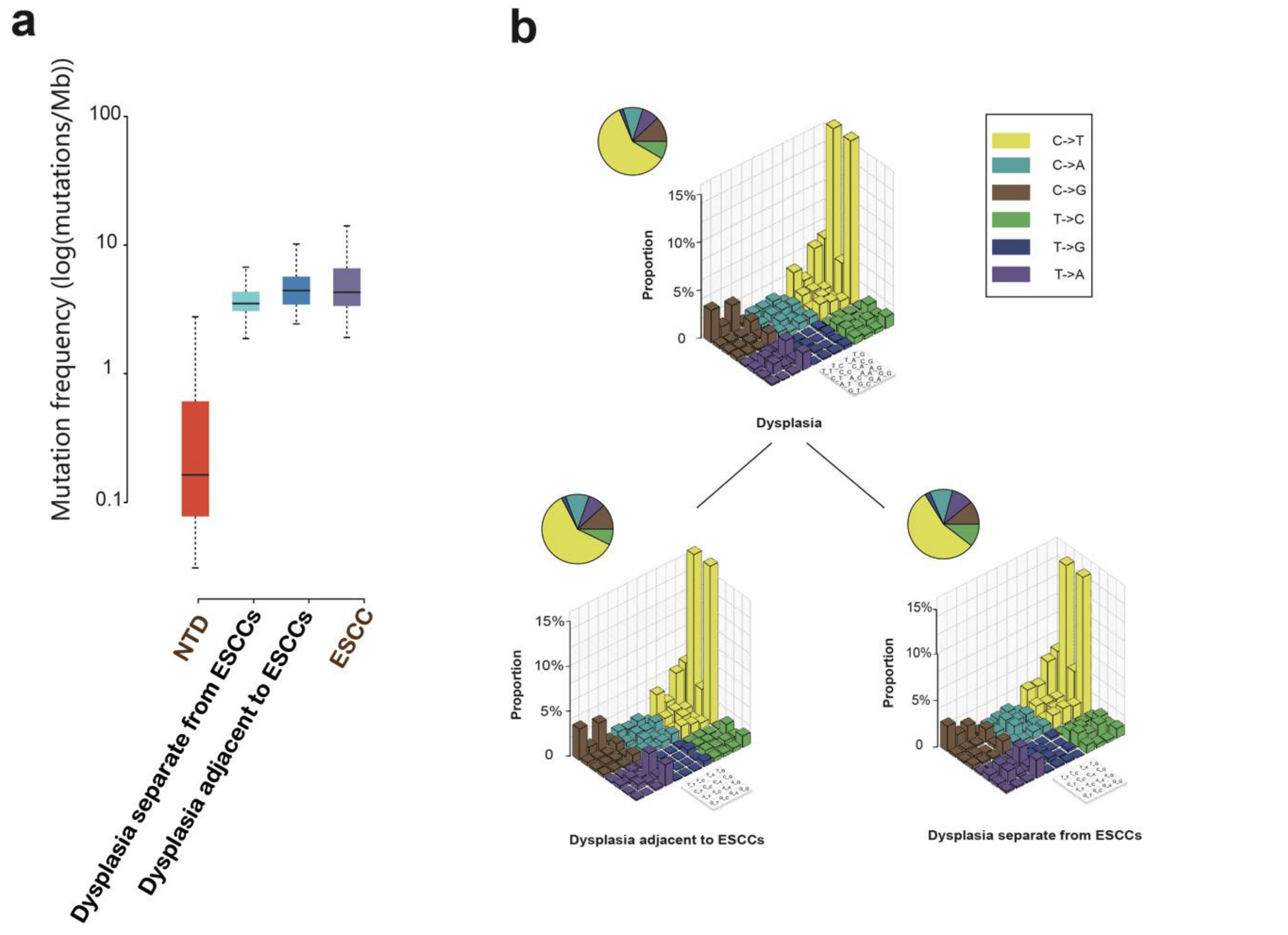

Supplementary Figure 2. Comparison of dysplasia samples adjacent to ESCCs and dysplasia samples separated from ESCCs. a) Comparison of the mutational density of all NTDs (n=13), dysplasia adjacent to ESCCs (n=47), dysplasia separate from ESCCs (n=28) and ESCCs (n=62). The y-axis is shown on a log10-transformed scale. **b)** 'Lego' plots displaying the frequency of base substitutions within specific trinucleotide mutational contexts in dysplasia samples. All the dysplasia samples (top) were from the TD cohort and divided into two categories: adjacent to ESCCs (left bottom) and separate from ESCCs (right bottom).

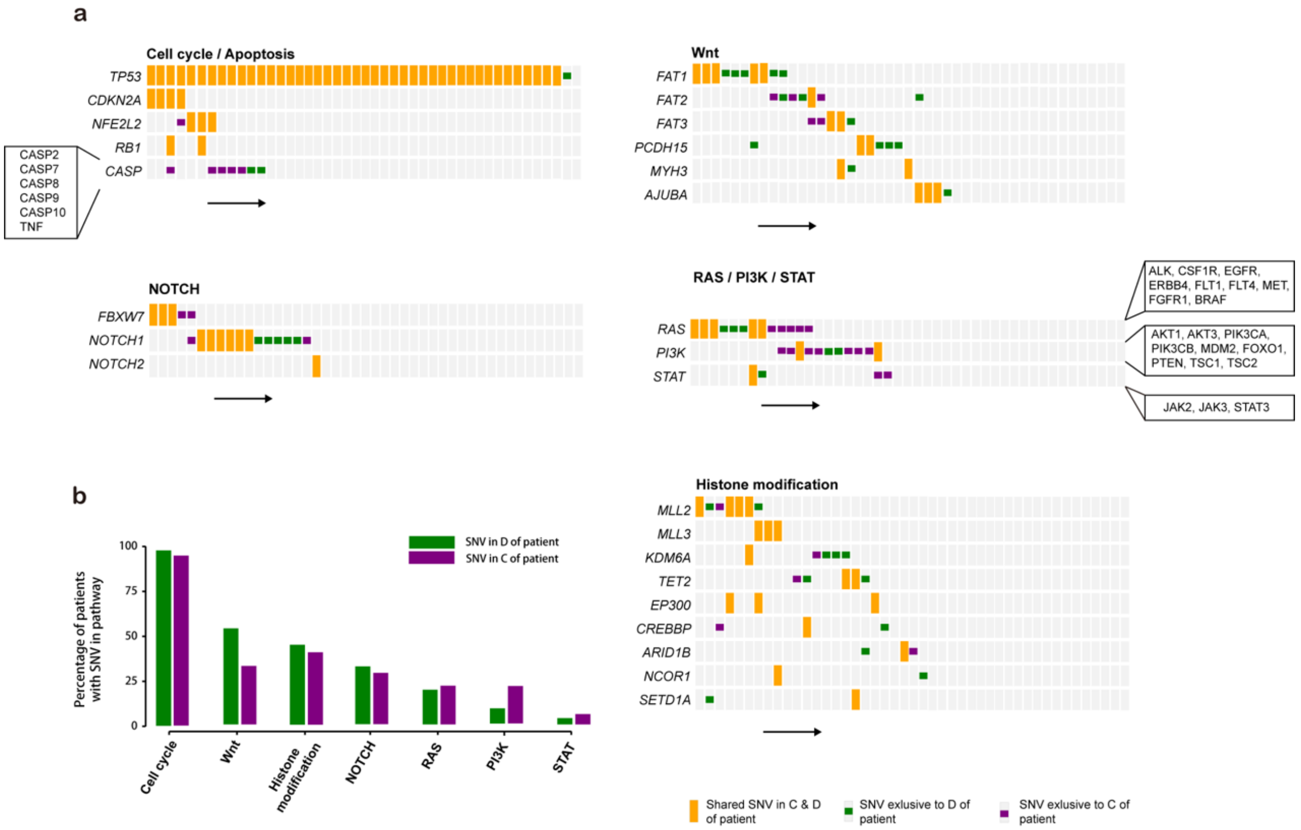

Supplementary Figure 3. Pathways affected by somatic mutations in dysplasia samples and ESCCs. a) Heat maps of the mutated genes in several ESCC-associated pathways. Patients are displayed in columns and arranged in a mutually exclusive pattern. The apoptosis, RAS, PI3K and STAT pathways are arranged in single rows, respectively, and the mutated genes are listed beside the heat maps. C, ESCCs; D, dysplasia samples. **b)** Bar plots comparing the frequency of altered pathways in dysplasia samples and ESCCs over patients. No statistical significance was observed (Fisher's exact test).

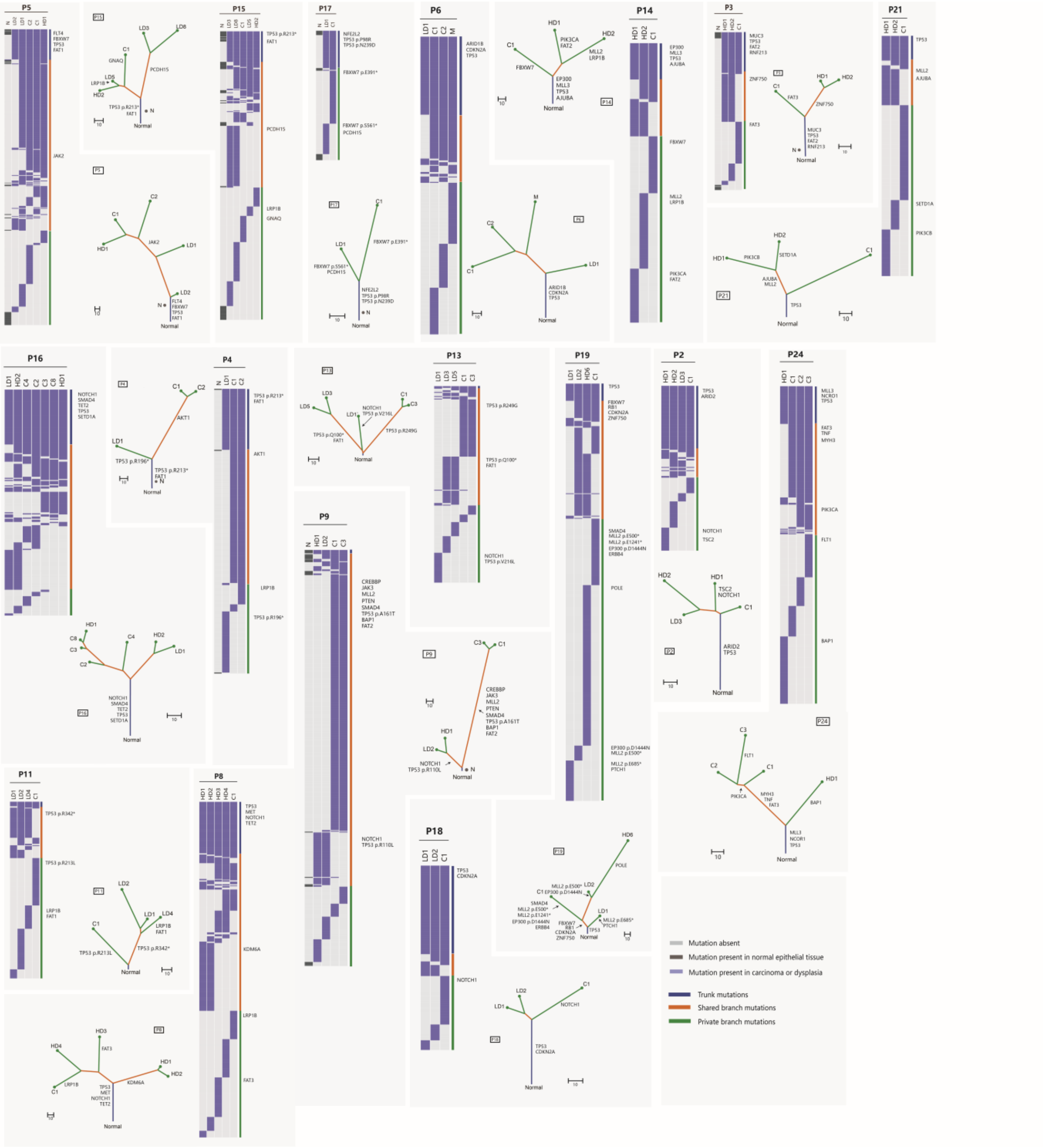

Supplementary Figure 4. Regional distributions of somatic mutations and phylogenetic trees of all the left cases. Heat maps showing the regional distribution of all mutations among different samples (normal epithelial tissue, dysplasia and carcinoma) in each case. Mutations are further divided into three categories: present in all samples (dark blue), present in more than one but not all samples (orange) and present in only one sample (green). Phylogenetic trees are constructed based on the maximum parsimony algorithm for each case. The color of each line is corresponding to the categories of mutations shown in the heat map. The length of the trunk and branch is proportional to the number of mutations in each sample.

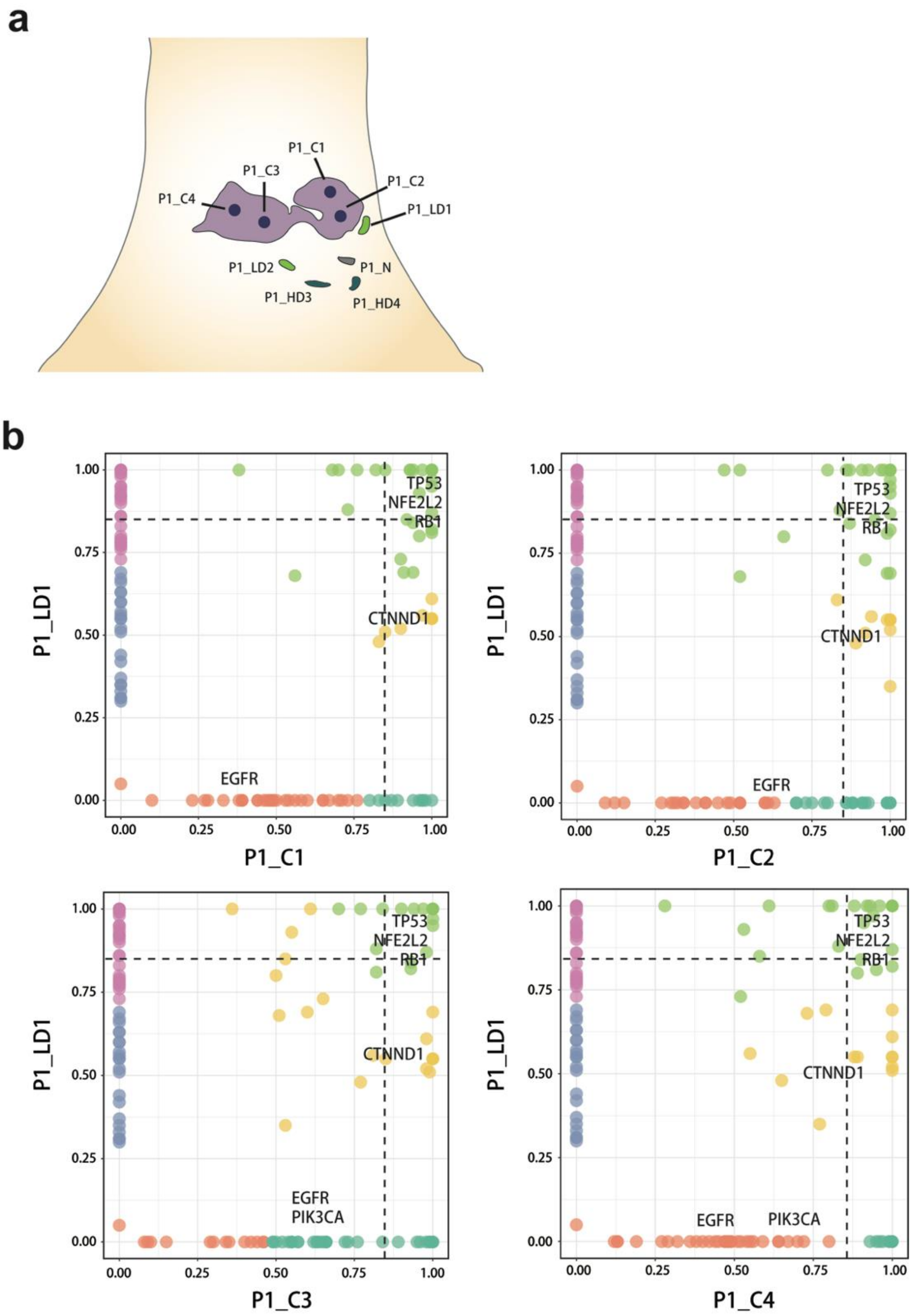

Supplementary Figure 5. Clonal relationship of one dysplasia sample and four ESCCs in P1. a) Geographical locations of all samples in P1. Histopathological types of all samples in P1 are indicated in different colors. **b)** Plots showing the cancer cell fraction (CCF) of mutations in LD1 against C1, C2, C3 and C4 in P1. The mutations are colored by different clusters. Putative driver mutated genes are indicated. The dash lines indicate the CCF value of 0.8.

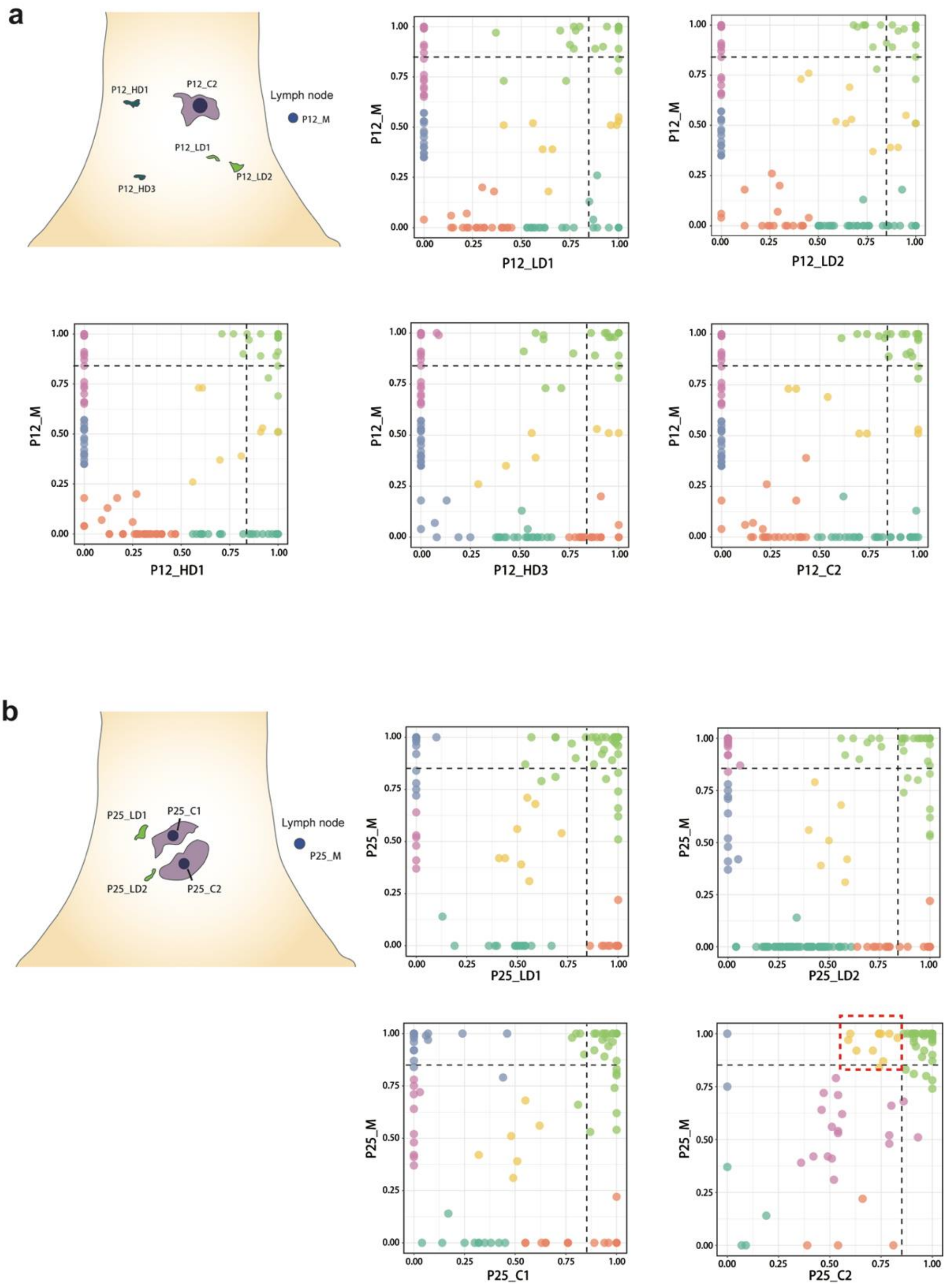

Supplementary Figure 6. Clonal relationship of the metastasis and other samples in P12 and P25. Geographical locations of all samples and plots showing the cancer cell fraction (CCF) of mutations in the metastasis sample against other samples in **a)** P12 and **b)** P25. The histopathological types of all samples are indicated in different colors, and the mutations are colored by different clusters. The dash lines indicate the CCF value of 0.8. The red rectangular in the scatter plot of P25_M versus P25_C2 marks the mutation cluster that is subclonal in P25_C2 but clonal in P25_M.

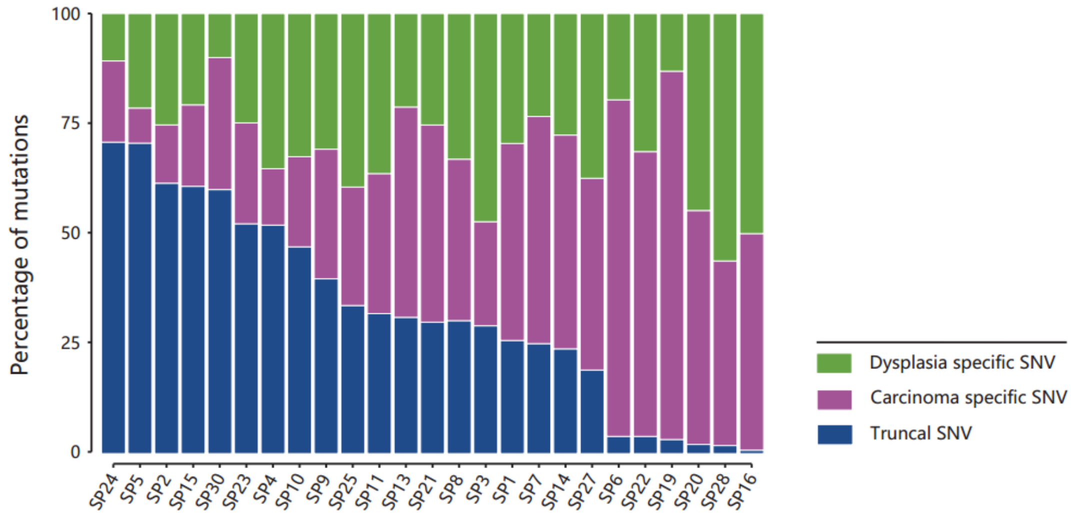

Supplementary Figure 7. Distribution of trunk and branch mutations for cases with paired samples. Graph indicating the proportion of trunk mutations (dark blue), carcinoma-specific mutations (purple) and dysplasia-specific mutations (green) in all 25 cases with paired samples. The ratio of trunk mutations ranges from 0.7% to 70.7%, with a median value of 29.9%.

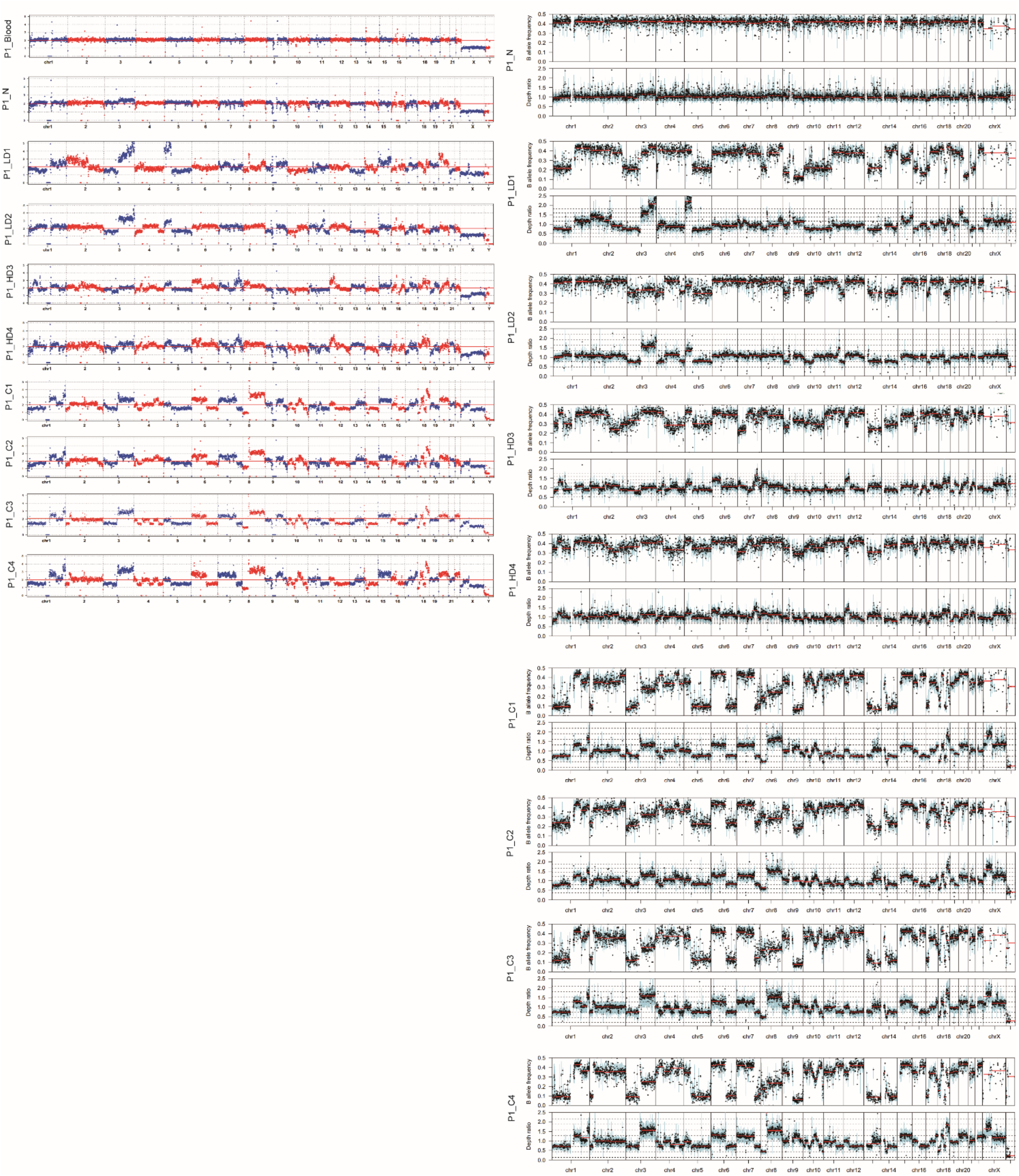

Supplementary Figure 8. Copy number states of samples in P1 decrypted by low-depth whole genome data and exome data. Left, plots showing the copy number states evaluated by low-depth whole-genome sequencing data. Horizontal axis corresponds to the position of genome. Copy number of windows with 500Kb in size are indicated. Right, plots showing the copy number states evaluated by whole exome sequencing data. Red lines represent the fitting copy number of the segments. Dashed lines mark the integer value of copy number.

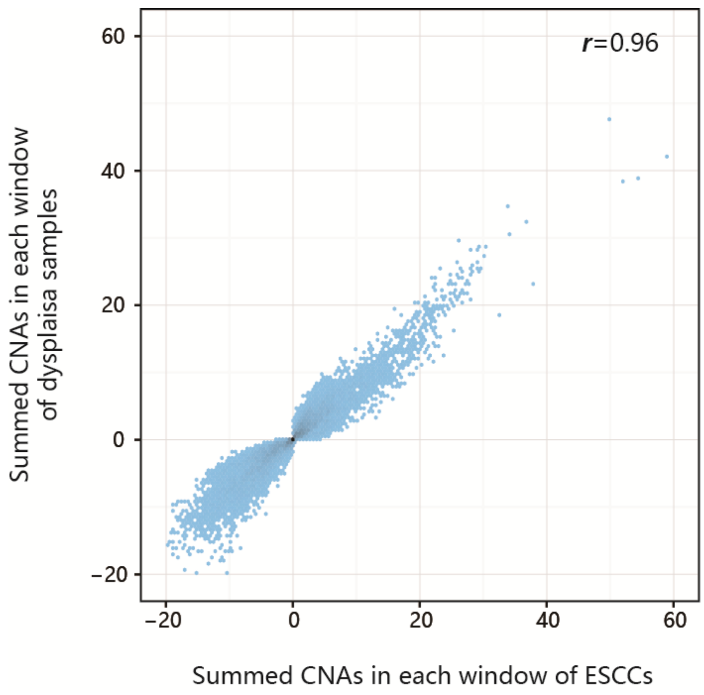

Supplementary Figure 9. Correlation of summed copy number alterations between dysplasia samples and ESCCs. The scatter plot showing the correlation of copy number alterations between 33 dysplasia samples and 33 ESCCs with low-depth whole genome sequencing data. Each dot represents a 500Kb-window, in which the depth ratio of all samples are transformed with log2 and summed. *r*, Pearson correlation coefficient.

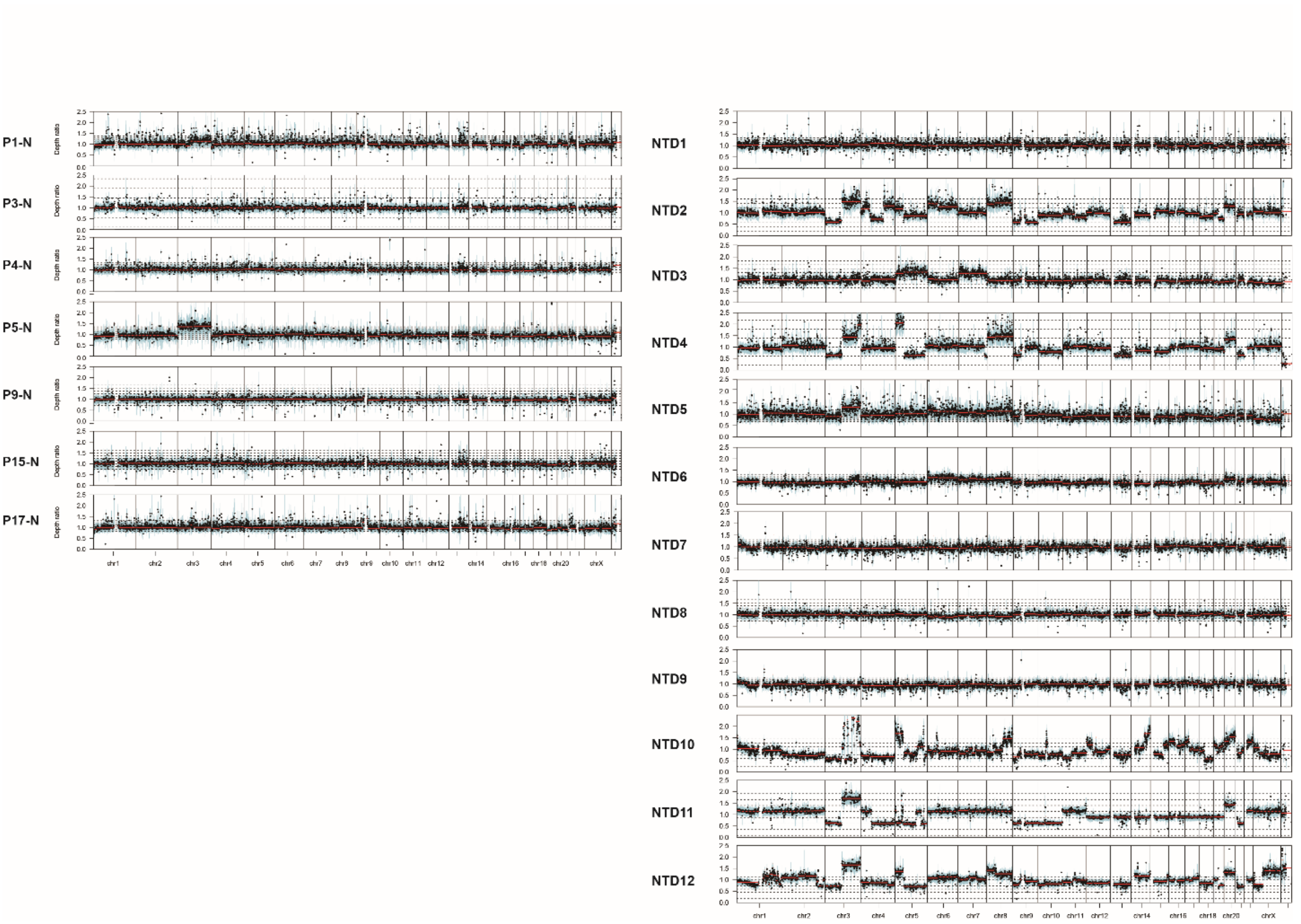

Supplementary Figure 10. Copy number states of non-tumor dysplasia and morphologically normal epithelial tissues. Diagrams showing the copy number states of 7 morphologically normal epithelial tissues (left) and 12 NTDs (right) evaluated by whole exome sequencing data. Red lines represent the fitting copy number of the segments. Dashed lines mark the integer value of copy number.

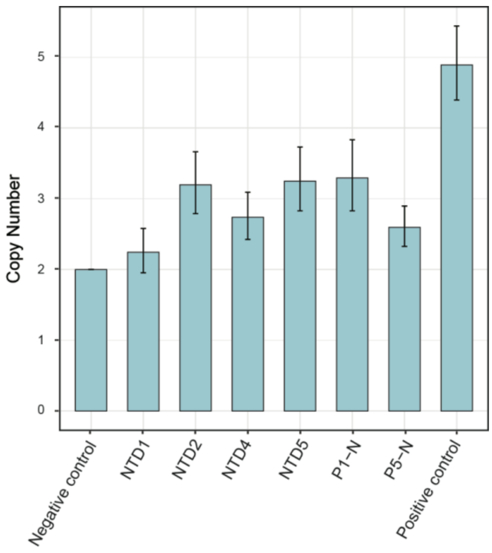

Supplementary Figure 11. Validation of amplification in chr3q by qPCR. Bar plots showing the copy number state of 3q26.3 validated by qPCR of 4 NTDs and 2 normal epithelial tissues. gDNA extracted from 5 blood samples were used as negative control and gDNA extracted from 3 ESCC samples with amplifications in 3q26.3 were used as positive control. The experiments of each sample were repeated 3 times. The 95% confidence interval is indicated.

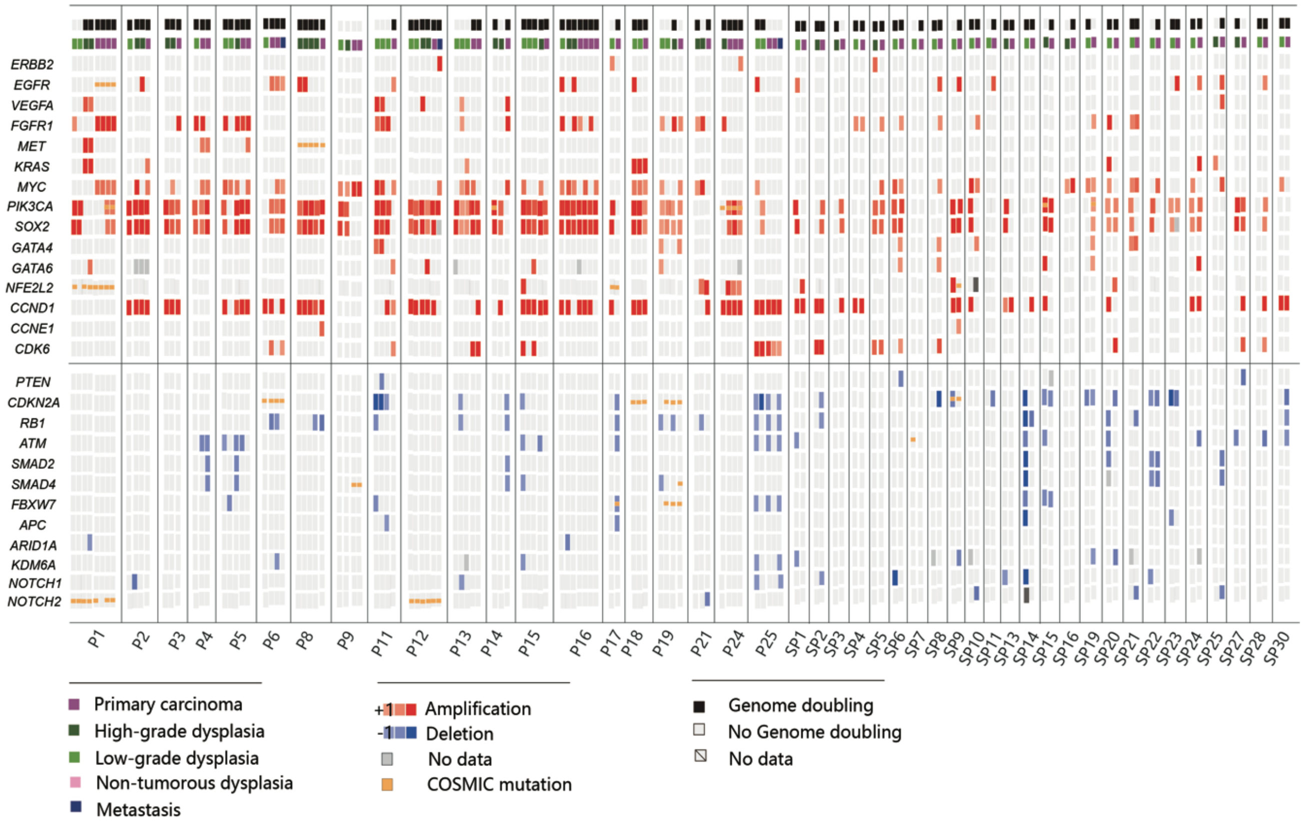

Supplementary Figure 12. Comparison of amplifications and deletions of key driver genes in individual patient. Plots showing the amplification of oncogenes (red), deletion of tumor suppressor genes (blue) and COSMIC mutations (orange) in each patient. The genome doubling status and histopathological types of the samples were marked on the top.

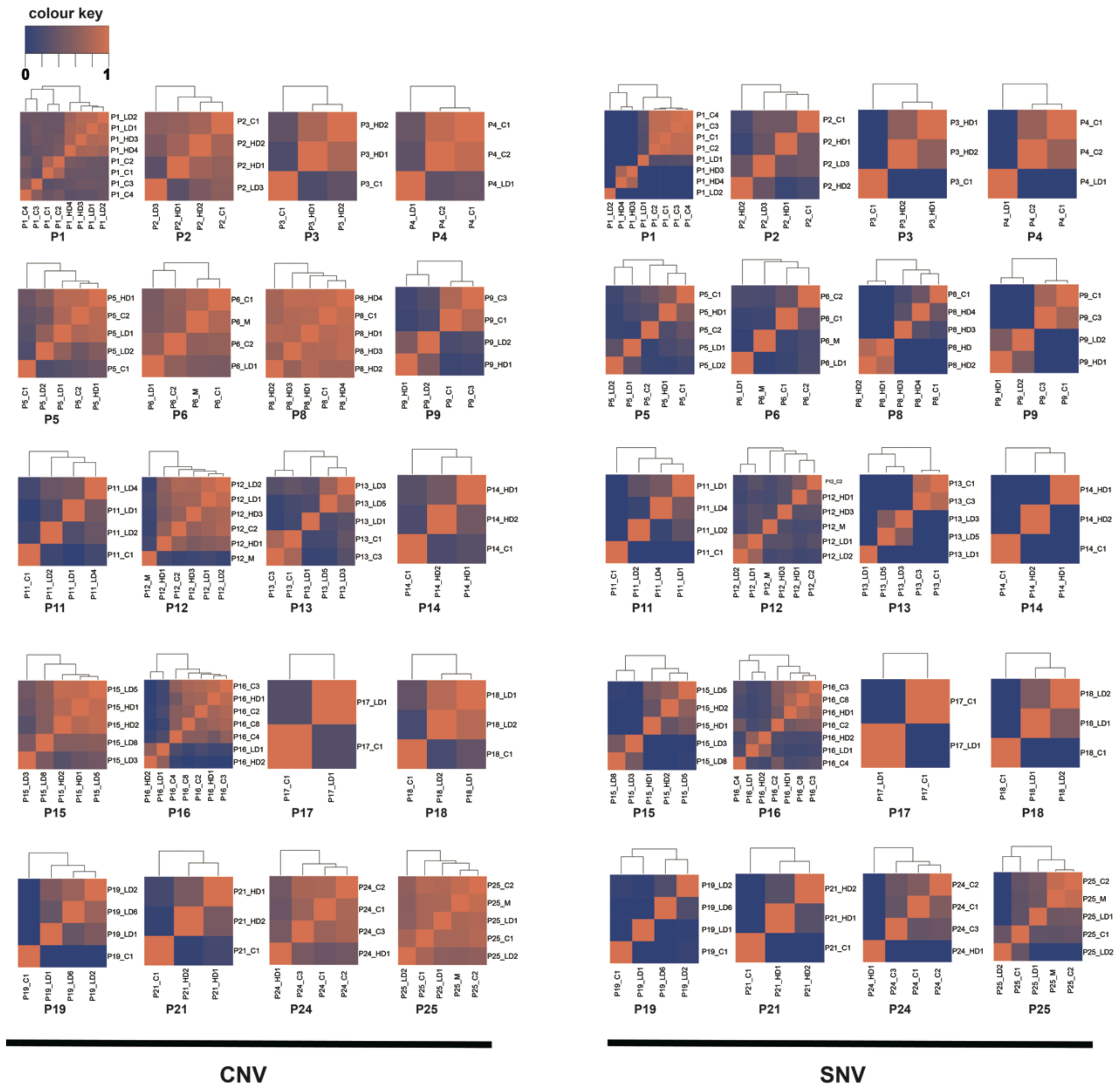

Supplementary Figure 13. Correlation of different samples in each case. Pearson correlation coefficients are calculated based on the SNV file and depth ratio file of samples in each case. Values of coefficient from low to high are colored from dark blue to orange.

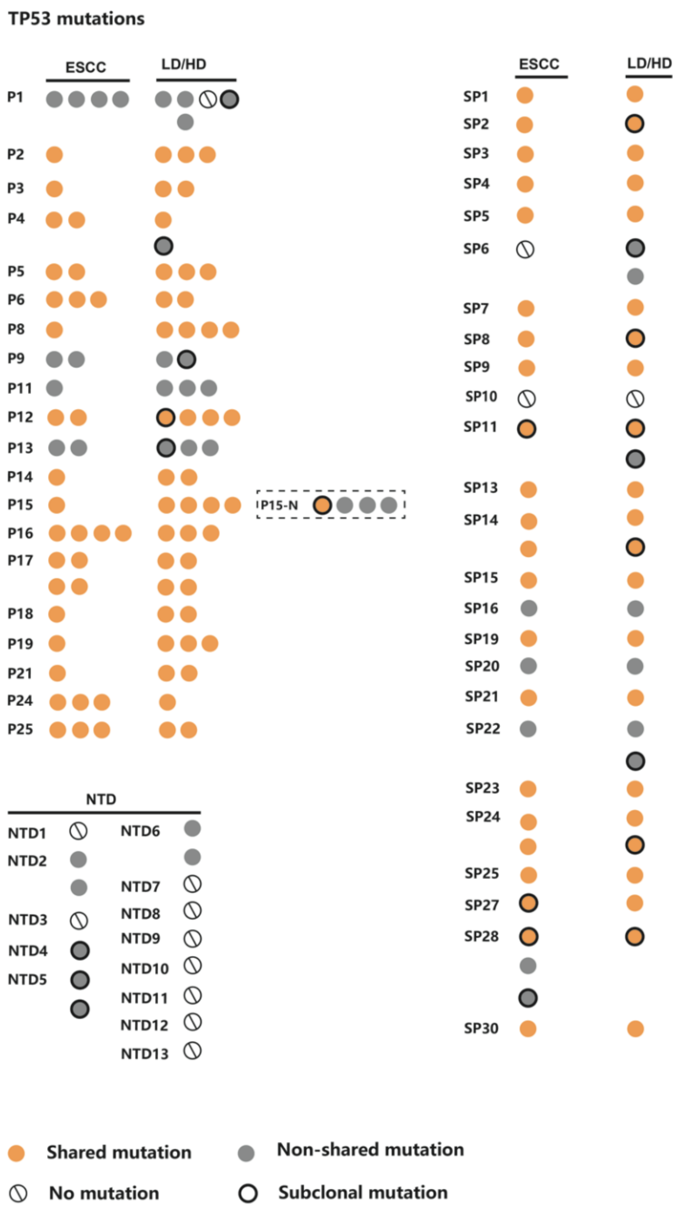

Supplementary Figure 14. Summary of *TP53* mutations in each case. Graph displaying the number and the status of whether being shared of *TP53* mutations in each case. Case ID is labelled on the left. In each case, dysplasia samples and ESCCs are separated. Every dot represents a mutation in one sample. If a case has multiple samples, dots are arranged horizontally. If a sample has two or more different mutations in *TP53*, dots are arranged vertically. Mutations in *TP53* shared by all samples of each case are marked as orange, and those mutations not shared by all samples are marked in gray. Subclonal mutations are annotated using black circle. One normal epithelial tissue, P15-N, which possesses 4 mutations in *TP53* is indicated.

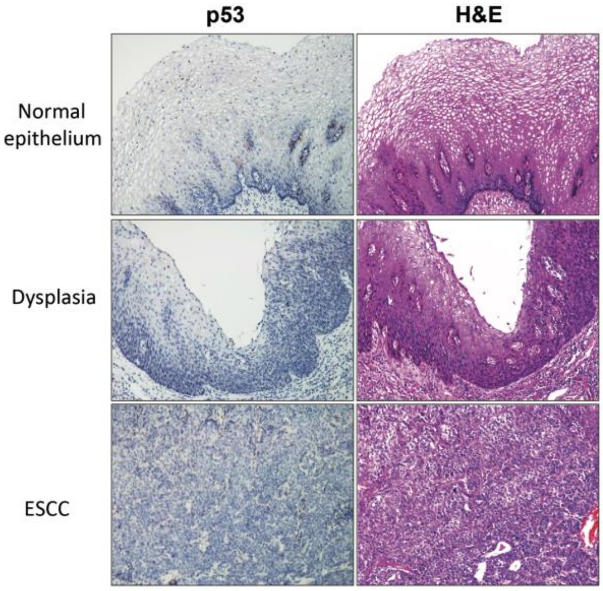

sample name	gene	mutation effect	a.a. change
P4_C1	p53	STOP_GAINED	p.R213*
P4_LD1	p53	STOP_GAINED	p.R213*

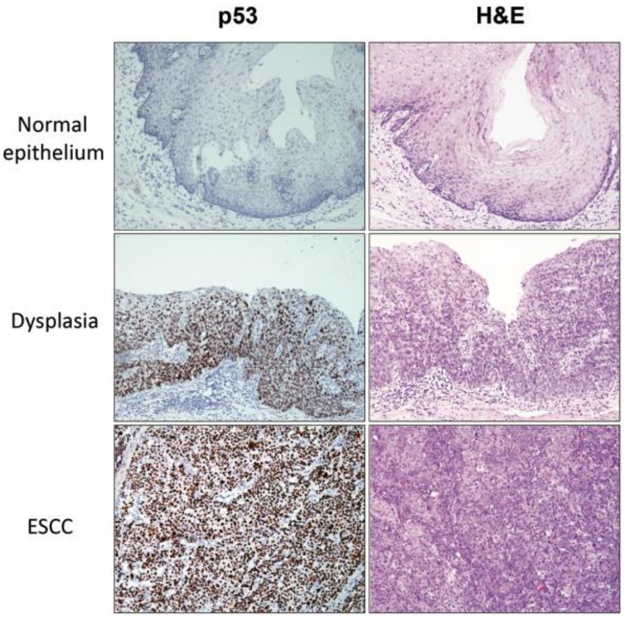

sample name	gene	mutation effect	a.a. change
P9_C1	p53	NON_SYNONYMOUS_CODING	p.A161T
P9_LD2	p53	NON_SYNONYMOUS_CODING	p.R110L

Supplementary Figure 15. The immunohistochemistry (IHC) staining of p53 protein and Haematoxylin & Eosin (H&E) staining of representative cases. IHC staining and H&E staining photos of p53 in normal epithelium, dysplasia and ESCCs from the case P4 and P9 showing the negative and positive staining of p53, respectively (100X magnification). The mutation information of these two cases is listed in the table below the IHC and H&E staining photos. a.a. change denotes amino acid change.

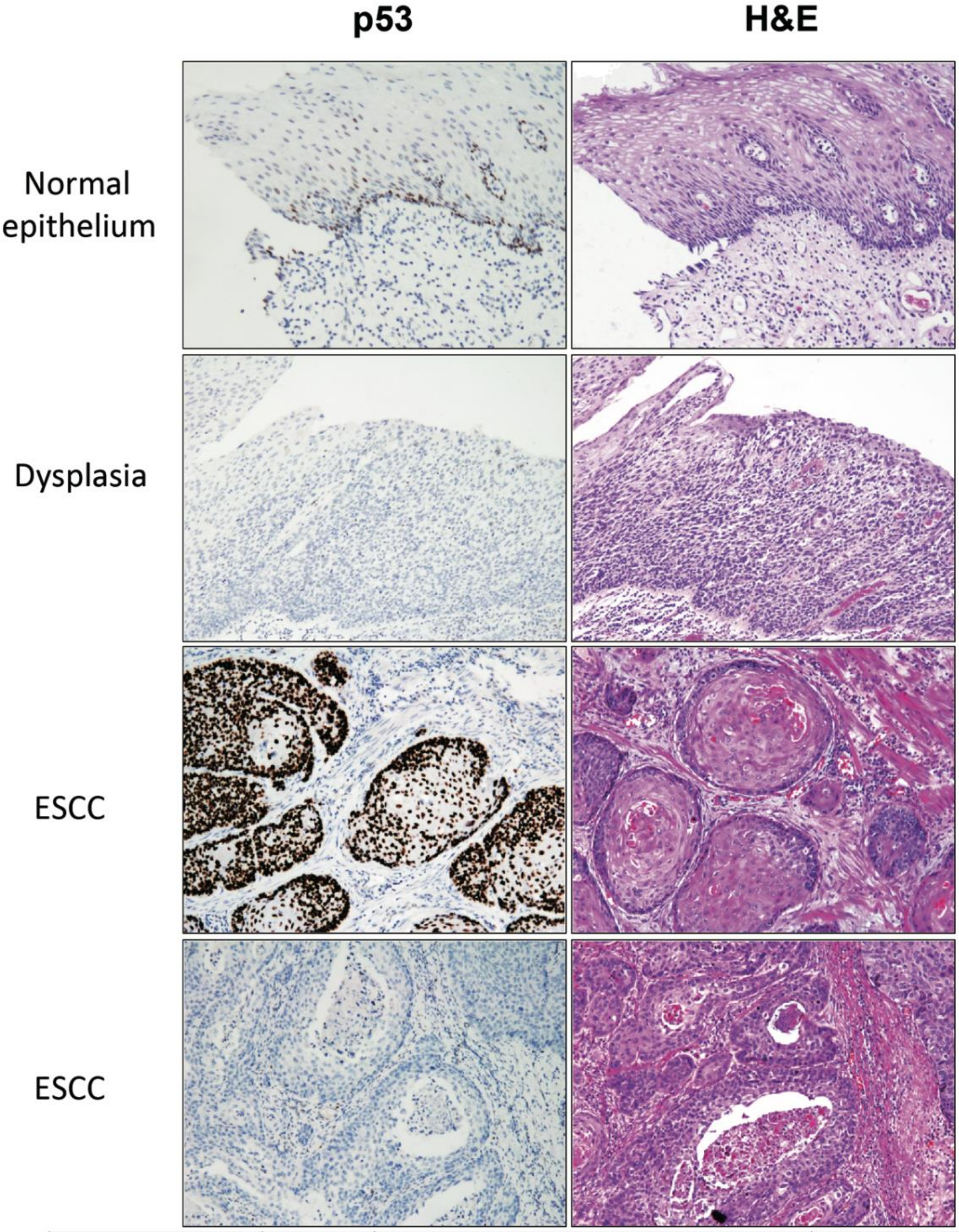

sample name	gene	mutation effect	a.a. change
SP11_C	p53	STOP_GAINED	E/*
SP11_C	p53	NON_SYNONYMOUS_CODING	R/H
SP11_LD	p53	STOP_GAINED	E/*

Supplementary Figure 16. The IHC staining of p53 protein and H&E staining of representative case. IHC staining and H&E staining photos of p53 in normal epithelium, dysplasia and ESCCs from the case SP11 showing the mix of both the negative and positive staining of p53, respectively (100X magnification). The mutation information of these this case is listed in the table below the IHC and H&E staining photos. a.a. change denotes amino acid change.

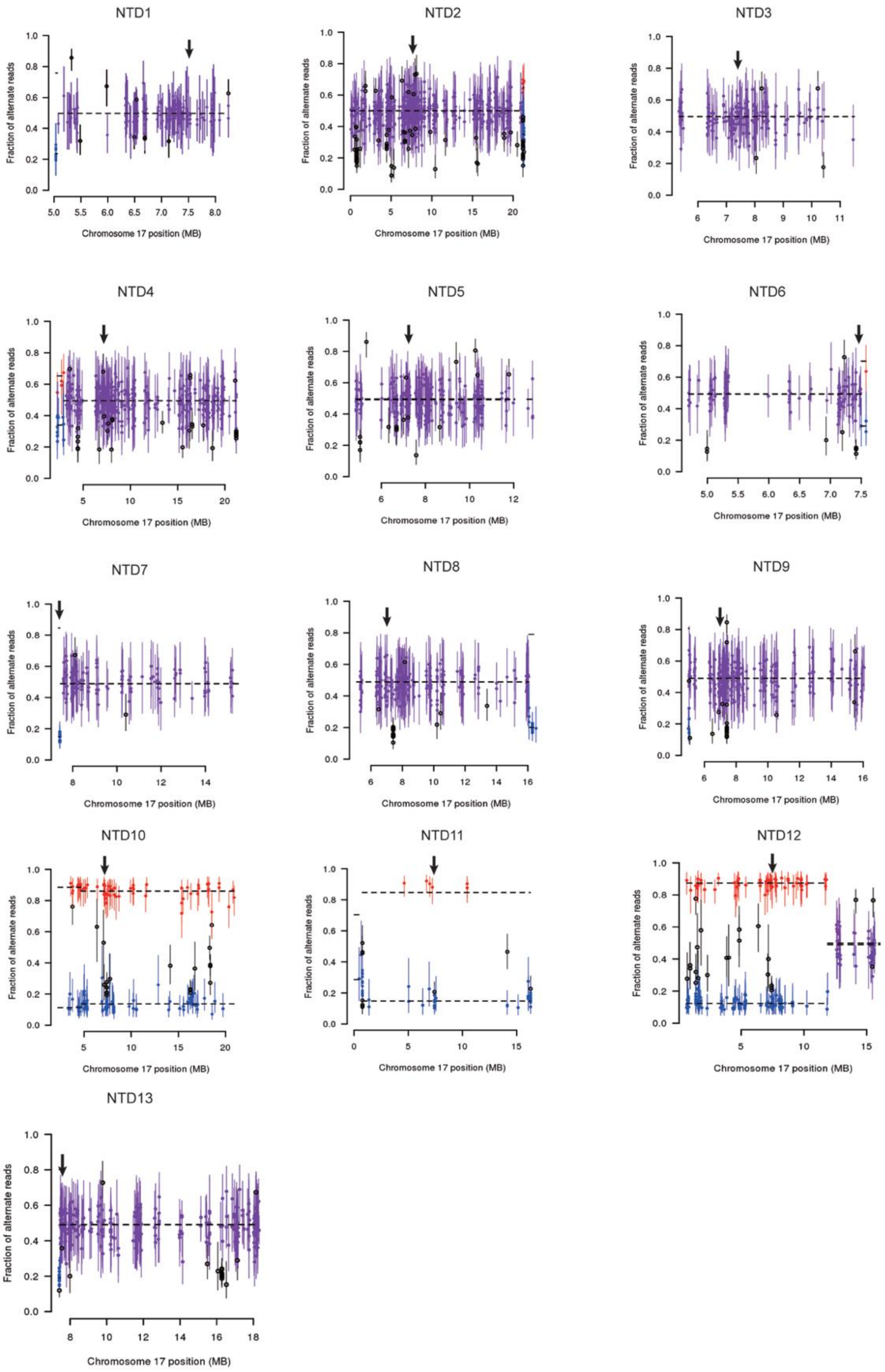

Supplementary Figure 17. The LOH states of chr17 in NTDs. Diagram displaying the fraction of alternative reads of heterozygous SNPs located within chr17. Chromosome regions with LOH (red/blue) and without LOH (purple) are indicated. The arrow refers to the positon of *TP53* in each case.
